# Vitamin C reduces the severity of common colds: a meta-analysis

**DOI:** 10.1186/s12889-023-17229-8

**Published:** 2023-12-11

**Authors:** Harri Hemilä, Elizabeth Chalker

**Affiliations:** 1 Department of Public Health, University of Helsinki, POB 20, Helsinki, FI FI-00014 Finland; 2 Biological Data Science Institute, Australian National University, Canberra, ACT 2601 Australia

**Keywords:** Antiviral agents, Ascorbic acid, Double-blind method, Randomized controlled trials, Respiratory tract Infections, Severity of Illness index, Treatment outcome, Vitamins

## Abstract

**Background:**

Randomized trials have shown that vitamin C shortens the duration of common colds. Some trials reported greater effects on severe cold symptoms compared with mild symptoms. This review systematically compares the effects of vitamin C on severe and mild common cold symptoms.

**Methods:**

We included all placebo-controlled trials of orally administered vitamin C in doses of at least 1 g/day for the common cold for people in good health at baseline. The analysis was restricted to trials which reported both the total duration of the common cold, and the severity of the common cold measured using severity scales, the duration of more severe stages of the cold, or proxies for severe colds such as days indoors. Findings were pooled using the inverse variance, fixed effect options of the metacont function of the R package meta to calculate the ratio of means estimate.

**Results:**

Fifteen comparisons from 10 trials which reported both mild and severe symptoms were identified. All trials were randomized and double-blind. Compared to placebo, vitamin C significantly decreased the severity of the common cold by 15% (95% CI 9–21%). The direct comparison of the effect of vitamin C on mild and severe symptoms was limited to five comparisons which found that vitamin C had a significant benefit on the duration of severe symptoms. In this subset, there was a significant difference in the size of the effect of vitamin C on the overall duration of colds versus the duration of severe colds (P = 0.002), and vitamin C had no significant effect on the duration of mild symptoms.

**Conclusions:**

The common cold is the leading cause of acute morbidity and a major cause of absenteeism from work and school. However, absenteeism is dependent on the severity of symptoms. The finding that vitamin C may have a greater effect on more severe measures of the common cold is therefore important. Further research on the therapeutic effects of vitamin C on the common cold should measure outcomes of differing levels of severity.

**Supplementary Information:**

The online version contains supplementary material available at 10.1186/s12889-023-17229-8.

## Introduction

Using antibiotics to treat a typical acute common cold episode is futile because almost all colds are caused by viruses. Overuse of antibiotics is also expensive and contributes to antibiotic resistance, a significant concern. Yet, results from surveys carried out in the USA found that about half of all common cold patients received antibiotics [1, 2]. Given this, alternative treatment options for the common cold have substantial public health relevance.

Vitamin C has various effects on the immune system [3–6] and the common cold can alter vitamin C metabolism such that vitamin C levels are temporarily decreased in leucocytes, plasma, and urine [7–9]. Thus, it is plausible that the administration of vitamin C may have an effect on the pathogenesis of the common cold.

There has been interest in vitamin C for the common cold since the middle of the 20th century [10–15]. In 1971, Linus Pauling carried out a meta-analysis of vitamin C and the common cold, including the four placebo-controlled trials available at that time. He found very strong evidence that vitamin C decreased morbidity caused by the common cold (P = 0.000022) [16–18]. Subsequent larger and methodologically more robust randomized controlled trials (RCTs) supported Pauling’s conclusions.

In our Cochrane review on vitamin C and the common cold, we found that regular vitamin C supplementation of ≥ 0.2 g/day shortened the duration of colds by 9.4% (P < 0.00001) [19, 20]. Nevertheless, despite the strong evidence indicating that vitamin C has physiological effects on the common cold, there is a persistent wide-spread belief that vitamin C if of no benefit. This notion is based on several flawed reviews and an erroneous analysis of one particularly influential randomized trial [18, 21–24].

While the available evidence shows that vitamin C can affect colds, the optimal doses and the size of maximal benefit are not known, although two controlled trials indicated that 6–8 g/day of vitamin C might be twice as effective as 3–4 g/day [23–27]. It is also plausible that the size of the effect of vitamin C on the common cold varies by the specific outcome. For example, the effect on a runny nose might be different from the effect on days off work or school.

Two large RCTs found that the effect of vitamin C on days “confined to house” and “days absent from school” was greater than the effect on mild common cold symptoms in the same trials [28–30]. Severe cold symptoms are a common cause of absenteeism. Therefore, the effect of vitamin C on common cold severity and on pragmatic measures of severity, such as days off, or days with severe symptoms, is much more important than the effect on mild symptoms.

In our Cochrane review we calculated the effect of vitamin C on common cold severity using the standardized mean difference (SMD) scale [19]. However, the effect estimate on the SMD scale is difficult to interpret meaningfully [31]. In this current study we estimate the effect of vitamin C on common cold severity using the relative scale which is much easier to interpret [32, 33]. We also compare the effect of vitamin C on the duration of mild common cold symptoms with the effect on severe symptoms in RCTs that reported both outcomes.

## Methods

### Inclusion criteria

Placebo controlled trials were included in our meta-analysis regardless of whether or not they were randomised. Trials of children and adults of either sex were eligible for inclusion. We investigated orally administered vitamin C in doses ≥ 1 g daily over a period of supplementation for people in good health at baseline. This enabled us to focus on the effect of regular vitamin C intake on colds that occur during the period of vitamin C supplementation. The minimum dose limit was set because there is evidence of a dose-response relationship in the gram range [23–27], and therefore doses < 1 g/day are less informative. We restricted our analysis to trials that reported the effect of vitamin C on common cold severity by one of the severity outcomes described below. This meta-analysis was not pre-registered.

### Outcomes

We focus on two outcomes:

1) Severity of the common cold: this includes (a) symptom measures on a severity scale, (b) pragmatic definitions of severe colds such as days indoors or days absent from school, and (c) the duration of more severe stages of the cold which in this study we refer to as “severe” symptoms.

2) The overall duration of the common cold, which we use as an approximation for mild symptoms, since the duration of severe symptoms is a rather small part of the overall duration. For example, in the placebo-group of the larger Ludvigsson (1972) trial [30], the mean days off school was 1.16 days, for the overall duration of symptoms of 5.67 days. Thus, the duration of mild symptoms would be 4.51 days (80% of the mean overall duration, see Additional file 2). Although in an ideal case the duration of mild symptoms could be calculated in this way, given the heterogeneity in the trials, the overall duration appears more appropriate as an approximation of mild colds in our analysis.

### Search for trials

For our Cochrane review in 2013 we thoroughly searched the literature prior to 2013 [19]. For this current analysis we searched for trials on vitamin C and the common cold published since 2013; see Additional file 1 for details. We excluded the Johnston (2014) trial [34], on the basis that a minimum of 5 mild symptoms were required to define an illness episode as a common cold, thereby confounding the comparison of duration of mild (overall) symptoms and severity. In addition, the trial included just 28 cold episodes. We excluded Himmelstein’s (1998) comparison of runners [35], because the dropout rate was very high and divergent with 73% (38/52) of participants dropping out in the placebo group compared with 42% (22/52) in the vitamin C group.

### Assessment of risk of bias in included studies

In our assessment of risk of bias, we assessed random sequence generation; allocation sequence concealment; blinding (participants, study personnel); blinding (outcome assessment); completeness of outcome data, selective outcome reporting; whether placebo was distinguishable from vitamin C; and contamination (see below for a discussion of over-exposure in the control group). We judged each item as being of high, low or unclear risk of bias as set out in the criteria and provided a quote from the study report and a justification for our judgement for each item in the risk of bias table in Additional file 1. Both authors independently assessed risk of bias; disagreements were discussed, and consensus achieved.

### Measures of treatment effect

In our analyses on the effect of vitamin C on common cold severity, we used the relative scale since it adjusts for the baseline variability caused by variations between the trials due for example to differences in viruses, patients, and outcome definitions [32, 33]. We used the ratio of means (RoM) to calculate the relative effect [32].

When comparing subgroups within the same trial, we used the mean difference (MD), since the differences described above do not apply within a trial and the MD estimate is more useful practically.

### Statistical methods

The second Anderson et al. (1974) study had eight trial arms [26]. Participants in six arms received vitamin C following different protocols, and two arms received placebo. One of the two placebo arms (#6) had statistically significant baseline differences (up to P = 0.00002) when compared with the six vitamin C arms; see table  16 in [18]. However, the six vitamin C arms and placebo arm #4 were consistent in terms of baseline data, and so the comparisons presented in this review are with placebo arm #4. In our analysis, three vitamin C arms are compared with the single placebo #4 arm so we divided the placebo arm #4 participants between the three vitamin C arms to avoid triple counting the placebo participants. Furthermore, Anderson reported that “a labelling error had occurred in two of the 176 batches” [26](p 33). The authors argued that this error was taken into account, but this is a further methodological concern with the 8-arm trial.

Some trials presented the mean outcome, but not the respective SD. In some trials the P value for the difference was reported from which the SD was calculated (see Additional file 2). Moreover, in our Cochrane review, we estimated that on average the ratio of SD/mean for common cold duration was 0.7 [19]. Therefore, for trials that did not report P-values, we conservatively imputed SD as the mean cold duration of the treatment group for each of those trials. The consequence of this is that on average we are putting slightly reduced weight on our estimates of effect for these trials with missing P values.

In several trials some participants had more than one cold. Such colds are correlated because they occurred in the same person. However, one study found that the duration of the third common cold episode was very weakly explained by the duration of the first and second common cold episodes (R² = 0.05) [36]. In most studies the average number of colds was fewer than three per person. We therefore considered it unlikely that the within-person correlation of cold duration would have an impact on our analysis.

Additional file 2 (spreadsheet) shows our calculations. We pooled the included trials with the *metacont* function of the R package *meta* [37–39], using the inverse variance, fixed effect option to derive an estimate of the percentage effect of vitamin C on the outcomes. As a sensitivity analysis, we also constructed forest plots of the severity outcomes using (1) a SMD scale, and (2) a random-effects meta-analysis (Additional file 1).

The Cochran Q test has been criticized [40], however, in the absence of a useful alternative we used it to assess statistical heterogeneity among the trials in the meta-analyses. We did not calculate the I^2^ statistic [40]. Two-tailed P values were used in this review.

## Results

### Description of the included trials

We identified 10 trial reports which described 15 separate comparisons relevant for our meta-analyses. The main characteristics of the included trials are summarized in Table 1 and they are described in more detail in the Additional file 1.

**Table 1:** Characteristics of included trials

Trial [ref] Country	Participants	Age mean (y)	Number of colds	Vitamin C dose (g/day)	Duration of the trial (months)
Ludvigsson (1977 L) [30] Sweden	Schoolchildren	9	1279	1	3
Pitt (1979) [41] USA	Marine recruits	19	1219	2	2
Anderson 1972 [28, 29] Canada	Adults	29	1170	1 + 3	3
Anderson 1974#3 [26] Canada	Adults	34	611	2	3
Anderson 1974#1 [26] Canada	Adults	34	581	1 + 3	3
Anderson 1974#2 [26] Canada	Adults	34	560	1	3
Ludvigsson (1977P) [30] Sweden	Schoolchildren	10	236	1	3
Carr (1981T) [42] Australia	Twin adults and adolescents together	25	164	1	3
Carr (1981 A) [42] Australia	Twin adults and adolescents apart	25	128	1	3
Miller (1977) [43] USA	Schoolchildren	12 ^1)^	118	1	5
Constantini (2011 F) [36] Israel	Adolescent female competitive swimmers	14	51	1	3
Ritzel (1961) [14, 15] Switzerland	Schoolchildren in a skiing camp	12 ^1)^	48	1	0.2
Constantini (2011 M) [36] Israel	Adolescent male competitive swimmers	14	47	1	3
Himmelstein (1998) [35] USA	Adults	44	26	1	3
Sabiston 1974 [44]	Marine recruits	25	20	1	0.2

1) Miller included 6–17-year-old children, but mean age was not published; 12 years is the middle of the range [43]. Ritzel’s trial included “school children in two skiing courses”, but age is not described; we assume age around 12 years [14]

Three trial reports including six comparisons measured the effect of vitamin C on days “confined to the house” and “absence from school”. The trials in this group included 2736 participants, with a total of 4437 common cold episodes observed during the follow-up (Table 1). In two trials, Anderson studied Canadian adults and asked persons not to enrol unless they normally experienced at least one cold during winter [26, 28, 29]. In the 1974 report, Anderson published findings for three vitamin C groups [26]. Ludvigsson first carried out a small pilot trial, and then a large trial with schoolchildren in Sweden [30].

Two small, short trials reported the duration of “severe symptoms”. The Sabiston and Radomski trial with Canadian military recruits in a Northern winter exercise reported “headache, chills and fever, general malaise, nausea or vomiting” [44]. The Ritzel trial with schoolchildren in a skiing camp in the Swiss Alps reported “muscle pain, headache, abdominal pain, vomiting, diarrhea, general feeling of sickness” [14, 15]. These two trials included 391 participants, with a total of 68 common cold episodes.

Five trial reports comprising seven comparisons described the effect of vitamin C on common cold severity using a severity scale. These trials included 975 participants, with a total of 1739 cold episodes observed during the follow-up period. Constantini et al. studied adolescent competitive swimmers in Israel [36], Pitt and Costrini studied US Marine recruits [41], Carr et al. studied Australian adolescent and adult twins [42], Miller et al. studied US school age twins [43], and Himmelstein et al. studied sedentary US adults [35].

The duration of the trials ranged from 2 to 5 months, except the two trials that reported severe symptoms, which ran for about a week [14, 44]. In eleven of the comparisons 1 g/day vitamin C was administered, 2 g/day was administered in two comparisons [26, 41], and in another two 1 g/day was administered regularly but the dose was increased to 4 g/day during the first 3 days of cold episodes [26, 28] (Table 1).

**Fig. 1:**
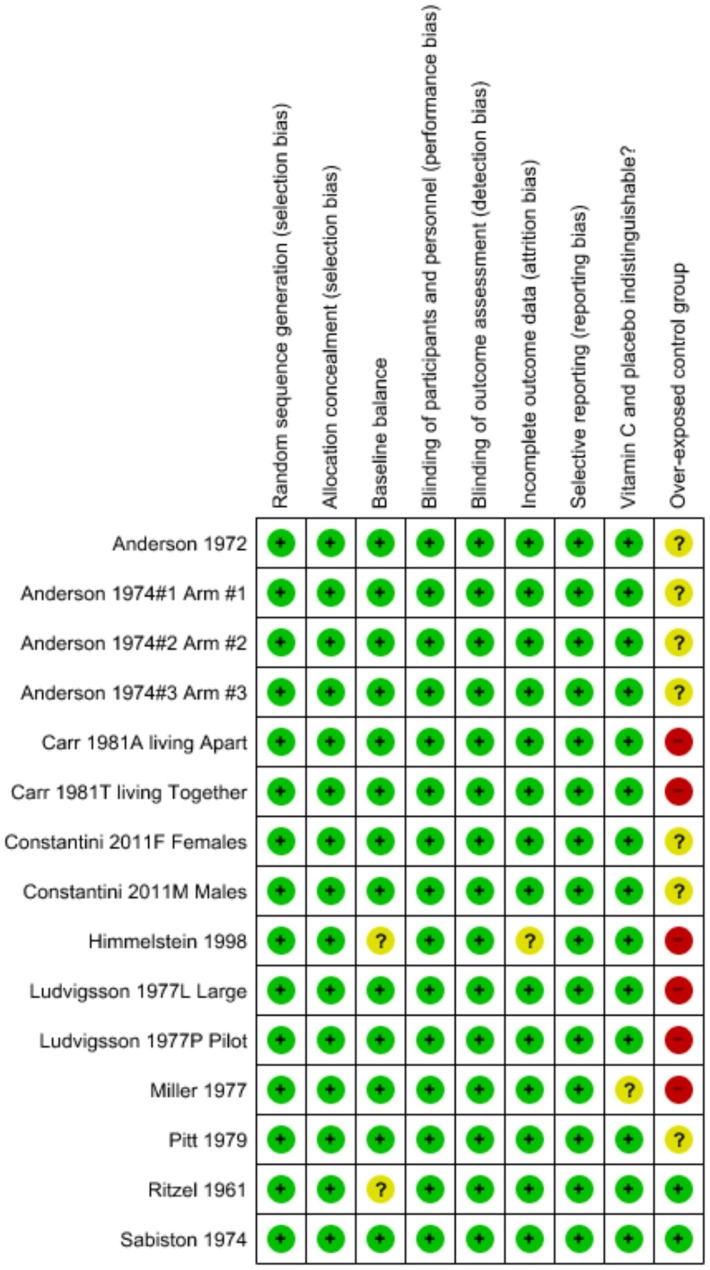
Risk of bias summary of the trials that reported on the effect of vitamin C on the common cold. Review authors’ judgments are shown for each risk of bias item for each included trial. A green plus sign (+) indicates that there is no substantial concern for bias in the particular quality item. A question mark (?) indicates that conclusions are unable to be drawn regarding potential bias. The red minus sign (-) indicates that there are concerns with the trial. The reference numbers to the trials are shown in Table 1. Justifications for the quality assessments are described in Additional file 1.

Our risk of bias analysis is shown in Fig. 1. All included trials were placebo-controlled as specified in the inclusion criteria, and all were randomized, though that was not one of our inclusion criteria. All trials were double-blind which also indicates that there was allocation concealment though that term was not used in the publications. The majority reported evidence of baseline balance in relevant variables and there is no indication that the rate of dropouts differed substantially between trial arms (see Additional file 1). Most trials stated that the placebo and vitamin C tablets were similar.

### Contamination

In some trials, participants in the control group receive some level of the same intervention as participants in the active treatment group. This is particularly so with vitamin C trials, and we refer to it as contamination. Contamination causes bias towards the null effect, so that the true effect may be greater than the observed effect. When contamination occurs, the control group is overexposed. For example, in the USA the recommended dietary vitamin C intake is 90 mg/day for men and 75 mg/day for women [45], and in the UK it is 40 mg/day [46]. Thus, in an ideal trial the dietary vitamin C intake in the placebo group should be 40 mg/day (UK) or 90 mg/day (US males). Doses for the placebo group which are higher than the recommendation can bias the effect of vitamin C supplementation towards the null effect. Contamination may occur when vitamin C was administered intentionally to the placebo group, through a particularly high dietary vitamin C intake, or through self-supplementation by people in the placebo group.

In seven comparisons we could not determine whether there was contamination (Fig. 1). In one trial there was unambiguously no contamination. Sabiston and Radomski wrote that “it was determined that the RP-4 rations … on which the men were living, apparently provided a maximum of 37–41 mg vitamin C per day in a single fruit-drink mix” [44]. In addition, Ritzel carried out his trial with schoolchildren in a skiing camp in the Swiss Alps in the early 1960s and we consider it unlikely that there was contamination although dietary vitamin C intake was not estimated [14, 15].

We found various levels of contamination in six of the included comparisons (Fig. 1). In the Miller twin study of children, vitamin C excretion in urine was high at baseline indicating high dietary vitamin C intake: on average 225 mg/day in the placebo group [43]. Furthermore, among boys in the placebo arm, urinary vitamin C excretion increased during the trial by 121 mg/day (P = 0.03) whereas the increase in girls was just 27 mg/day. Contamination might have occurred by boys swapping their tablets. In the Carr study, vitamin C was beneficial for twins living apart but not among twins living together [42], which may also indicate that tablets were swapped. Himmelstein et al. estimated that vitamin C intake in the placebo groups was on average 149 mg/day [35]. Finally, several studies administered vitamin C to the placebo group: Carr administered 70 mg/day [42], Miller et al. 50 mg/day [43], and Ludvigsson 30 mg/day in the pilot trial, and 10 mg/day in the large trial [30].

### Effect of vitamin C on common cold severity

**Fig. 2:**
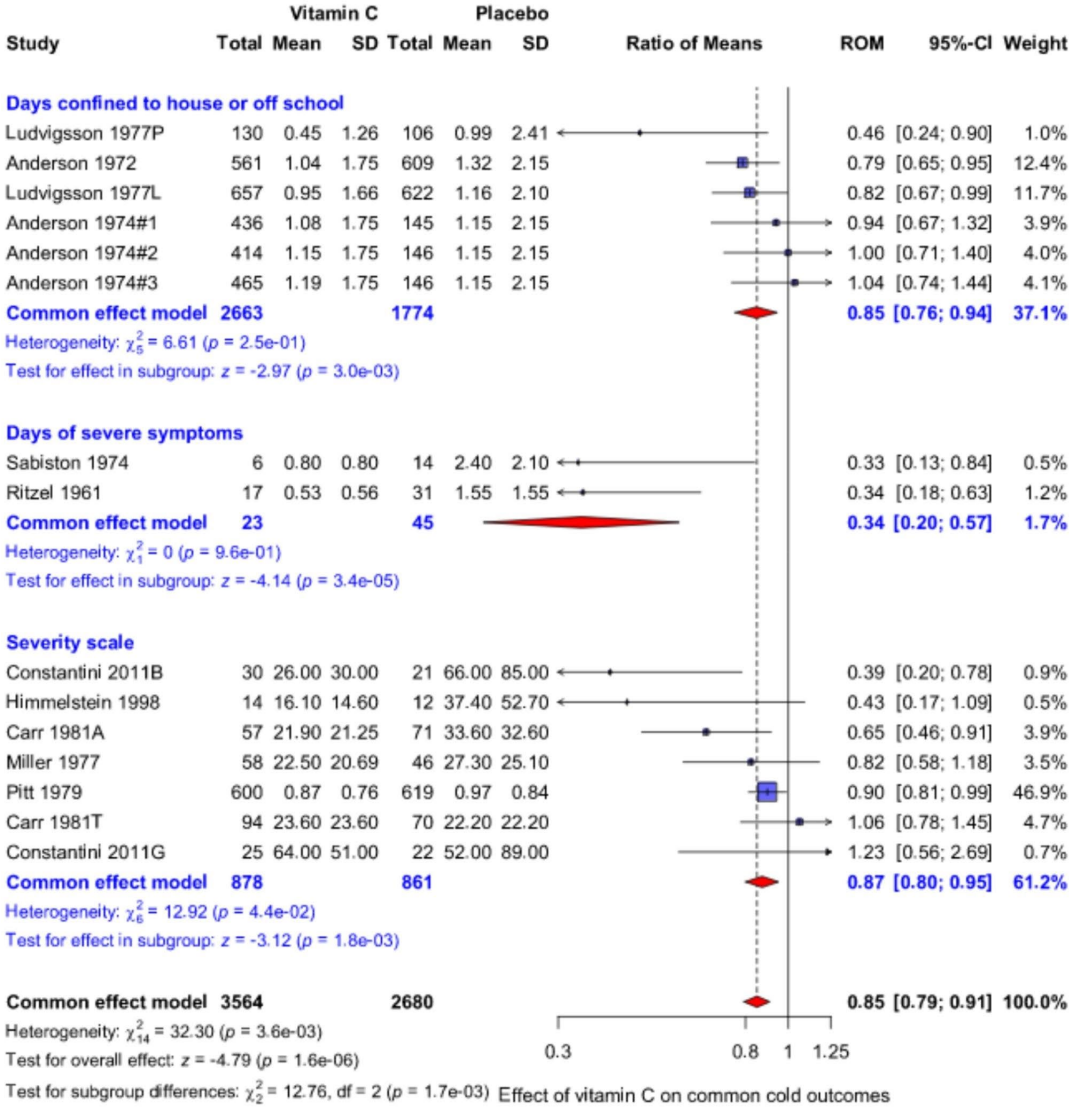
Effect of vitamin C on the severity of the common cold. The upper subgroup shows the effect on outcomes which are proxies for severe colds, the middle subgroup shows the effect on the duration of severe symptoms, and the lower subgroup shows the effect on severity scales. The horizontal lines indicate the 95% CI for the vitamin C effect and the blue squares in the middle of the horizontal lines indicate the point estimate of the effect in the particular trial. The size of the blue square reflects the weight of the trial in the meta-analysis. The red diamond shapes indicate the pooled effect and 95% CI for the three subgroups and for all 15 comparisons. See Additional files 1 and 2 for the description of the trials and the calculations. RoM, ratio of means; e.g. RoM = 0.8 corresponds to a 20% decrease in the outcome

Three trial reports with six comparisons contribute to the analysis of the effect of vitamin C on the days of “absence from school” [30] and days “confined to house” [26, 28, 29] (Fig. 2 top). On average, vitamin C shortened the duration of these outcomes by 15% (95% CI: 6–24%; P = 0.003). A statistically significant within-trial benefit was found in three comparisons. The larger Ludvigsson et al. trial with Swedish schoolchildren found that “absence from school” during common cold episodes was reduced in the vitamin C group by 18%. The 1972 Anderson et al. trial with Canadian adults reported a 21% reduction in days “confined to house” per episode.

Two short trials including participants under physical stress in cold climates reported the effect of vitamin C on the days of “severe symptoms” (Fig. 2 middle). The trial by Ritzel was with Swiss school children [14, 15], and the other by Sabiston with Canadian military recruits [44]. Both trials found about a 60% reduction in the duration of severe symptoms, but the confidence intervals are wide.

Seven comparisons contribute to the analysis on common cold severity on the severity scale (Fig. 2 bottom). On average, vitamin C decreased severity by 13% (95% CI: 5–20%; P = 0.002). Significant within-trial benefit was found in three comparisons. Constantini et al. found a benefit for male swimmers, but not for female swimmers [36]. Carr et al. found a benefit for twins living apart, but not for twins living together [42]. Pitt and Costrini found that vitamin C led to a 10% reduction in cold severity in US marine recruits [41].

The pooled effect of ≥ 1 g/day vitamin C across all 15 comparisons in Fig. 2 indicates a highly significant 15% reduction in common cold severity. Three large trials have a total weight of 71% in the meta-analysis: the 1972 trial by Anderson et al. [28], the larger trial by Ludvigsson et al. [30] and the Pitt and Costrini trial [41]. In a sensitivity analysis, we excluded these trials, but the estimate of reduction in common cold severity was not substantially changed. In the remaining trials covering just 29% of the total statistical weight, there was a 19% (P = 0.001) decrease in severity (see Additional file 1). Thus, the conclusions are not dependent on the three largest trials which have most influence on the analysis.

### Comparison of the effect of vitamin C on mild and severe symptoms of the common cold

**Fig. 3:**
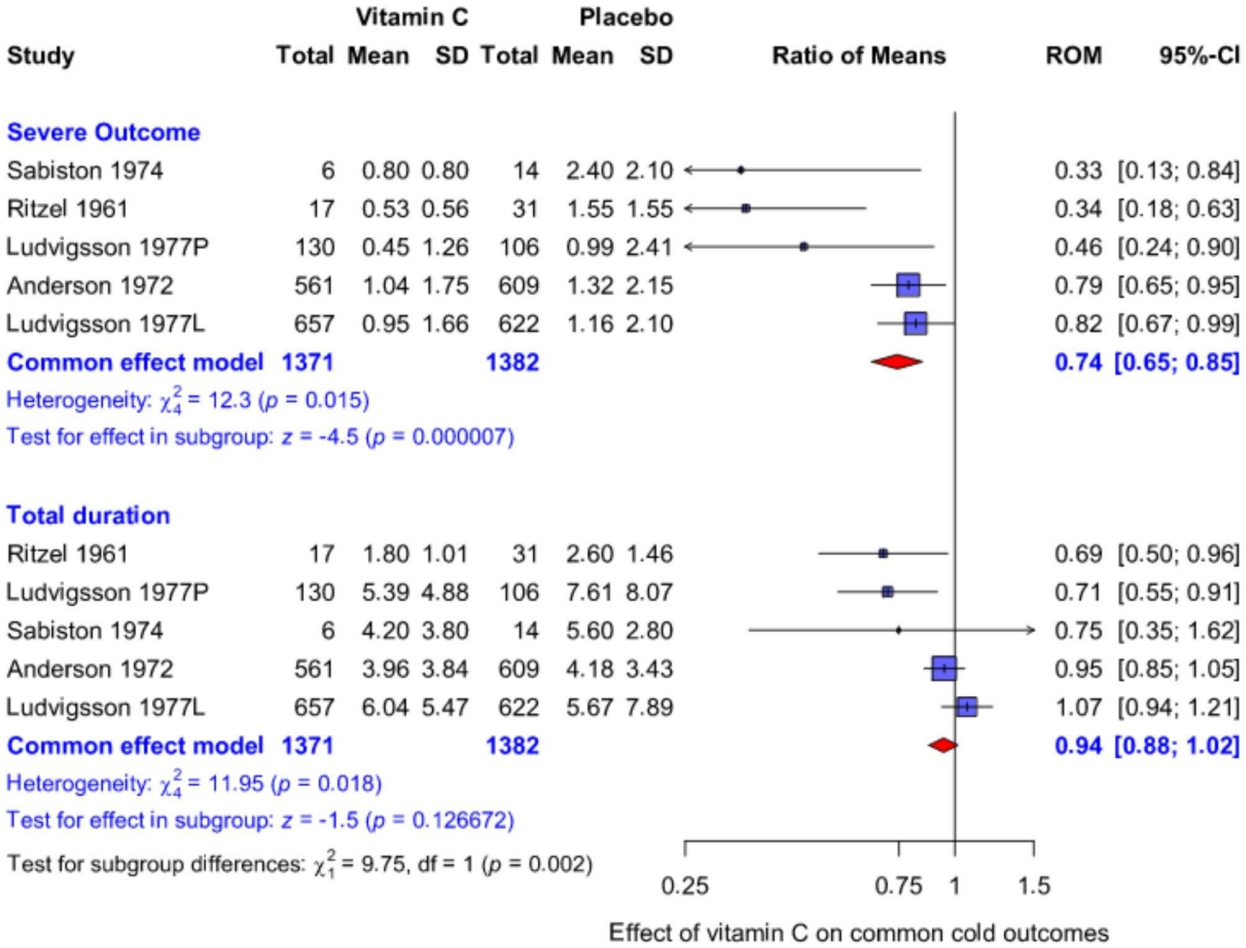
Comparison of the effect of vitamin C on mild and severe symptoms of the common cold. The upper subgroup shows the effect on the duration of severe outcomes and the lower subgroup shows the effect on the overall duration of any symptoms. See details in Fig. 2

Five trials found significant benefit from vitamin C on the duration of severe episodes of the common cold. In total, there were 2753 common cold episodes in these trials. We analysed these trials to determine whether vitamin C had different effects on the durations of mild and severe common cold symptoms. We found that vitamin C had a significant effect on the duration of severe symptoms (Fig. 3). Although there was strong evidence of a 26% reduction in the more severe measures of common cold, there was no evidence of effect on mild common cold symptoms with a narrow confidence interval. A test for the difference between the two outcomes in the same trials gives P = 0.002, though this P-value needs to be considered with some caution as the duration of severe and overall colds are not independent observations.

In Fig. 3, we did not include the trials that reported severity on a severity scale, since the measurement of severity is more ambiguous than the measurement of duration. For example, Pitt and Costrini reported that “each cold was rated by the recruits as being mild, average, bad, or the worst ever, and these four subjective classifications were given numerical ratings from 1 to 4” [41]. However, we should not assume that increases in a severity score from 1 to 2, and from 3 to 4 are equidistant in the same sense as an increase in the absence from school from 1 to 2 days, and from 3 to 4 days. Nevertheless, in a sensitivity analysis we included all 15 comparisons, and a significant difference remained in the effect of vitamin C on severe vs. mild symptoms of colds with P = 0.037 for the test of difference between the two outcomes (see Additional file 1).

### Subgroup differences in the effects of vitamin C

**Fig. 4:**
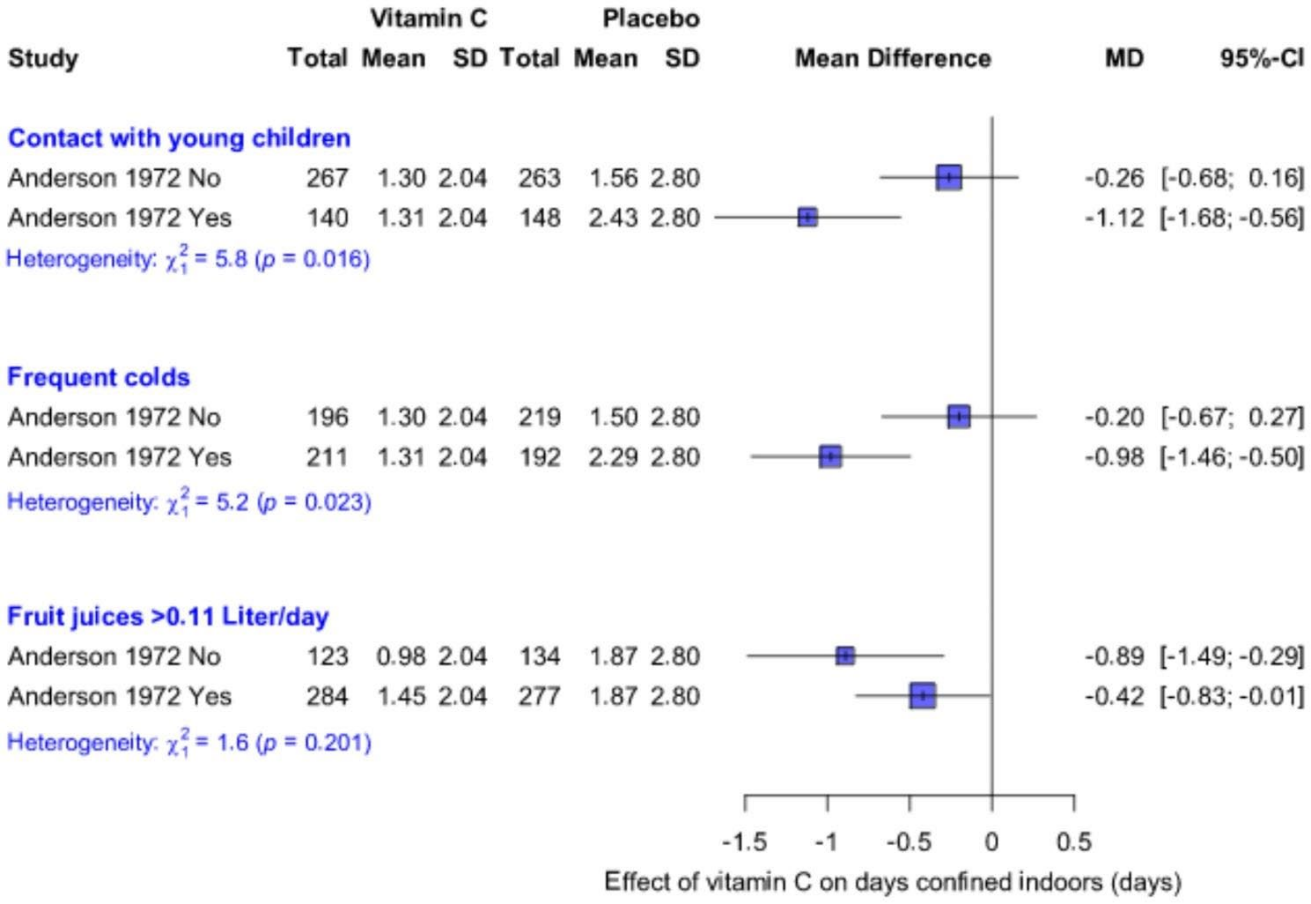
Effect of vitamin C on the total days confined indoors by subgroups in the Anderson (1972) trial. The scale of days is used in this figure since that is more useful in practical terms, and the population is homogeneous with respect to viruses, participants, and outcome definitions. For example, over the 3-month follow-up in participants who had contact with young children, vitamin C decreases days confined indoors by 1.12 days, and in those who did not have contact with children by 0.26 days. Thus, the effect was 0.86 days greater in the former (P = 0.016 for the difference in the effect between the subgroups). MD, mean difference

For our pooled analysis in Fig. 2 we assumed that vitamin C had a uniform effect within the three groups, and over all the included trials. This is a simplified assumption for the ease of calculation, but it is unlikely that a uniform effect exists across all population groups, even when using the same outcome definition. The 1972 Anderson et al. trial supports the notion that the effect of vitamin C can differ substantially between population groups [28, 29]. It is one of the largest common cold trials that has been carried out with a recorded 1170 episodes of illness. Days “confined to house” per episode was 21% shorter in the vitamin C group (Fig. 2). In addition, the proportion of participants who were not “confined to the house” during the trial decreased by 9.6 percentage points in the vitamin C group (47.4% vs. 57.0% P = 0.006; see Additional file 2). Together these effects contribute to the 30% reduction in days “confined to the house” per person (P = 0.001) [28]. Such a strong effect enables the comparison of clinically relevant subgroups (Fig. 4).

Anderson et al. reported that over the 3-month follow-up, vitamin C decreased the total number of days “confined to house” by 1.12 days in participants who had contact with young children, but by just 0.26 days in participants who did not have contact with young children (Fig. 4). This 0.86-day difference in the effect of vitamin C between the two subgroups is significant. They also reported that vitamin C decreased total days “confined to house” by 0.98 days in those who usually had ≥ 2 colds per winter, but by only 0.20 days in those who usually had 0–1 colds per winter. This 0.78-day difference in the effect of vitamin C is also significant. Anderson et al. also reported subgroup analysis by low vs. high intake of fruit juices, which is an important daily source of vitamin C. The benefit of vitamin C was more evident for participants who had low juice intake.

## Discussion

The term ‘the common cold’ does not denote a precisely defined disease and the symptoms and length of illness vary from person to person and from cold to cold [47]. Nevertheless, this illness is familiar to most people and can be readily self-diagnosed. Typical cold symptoms include combinations of nasal discharge and obstruction, sore throat, and cough, but it can also cause more severe symptoms. Sabiston and Radomski noted “it is these [more severe] symptoms which, in a civilian population, could predispose a person to remain at home” [44]. The common cold is the leading cause of acute morbidity and visits to physicians in high-income countries, and it is a major cause of absenteeism from work and school. In one analysis, the economic burden of the common cold was comparable to hypertension and stroke [48].

In common cold trials, explicitly defined outcomes (based on the duration and set of symptoms for example) are important to enable the measurement of identical comparisons in the trial groups. However, such definitions are biologically arbitrary. It is not possible to determine the cause of a runny nose or sore throat on the basis of symptoms, as there is no particular duration or combination of symptoms which point to either viral infection, allergy, or some mechanical irritation of the airways. Anderson et al. (1972) wrote that “we found it difficult to arrive at a single, generally acceptable definition of a ‘cold’, other than that the episode of illness should at some time be marked by symptoms in either nose or throat. The average frequency and total duration of ‘colds’ per subject were therefore calculated according to four different definitions of a ‘cold’ ” [28].

A few common cold trials reported that vitamin C seemed to have a greater effect on more severe forms of the common cold, so we systematically compared the effect of vitamin C on mild vs. severe symptoms of colds. In this study, we included trials that reported severity scores, duration of severe symptoms, or pragmatic outcomes such as days off school or days confined to the house. Over these three classes of outcomes, we found a 15% average decrease in the severe forms of the common cold in people being administered vitamin C (Fig. 2).

Thereafter, we directly compared the effect of vitamin C on mild vs. severe common cold outcomes by restricting our analysis to trials that reported statistically significant effects of vitamin C on the duration of severe forms of the common cold. Most of the non-significant findings were from small trials that had wide confidence intervals and were less informative (Fig. 2). We also excluded the Anderson et al. (1974) trial [26], based on concerns about the baseline balance [18], and errors in labeling (see Methods). The effect of vitamin C was significantly different on the mild vs. severe common cold outcomes (Fig. 3). Over the 5 included trials, vitamin C decreased the duration of severe common cold outcomes by 26% but had no effect on mild symptoms.

Some previous meta-analyses on medical conditions other than the common cold have also found that vitamin C may have greater effects on patients with more severe conditions compared with mild conditions [49–51]. Furthermore, two RCTs [28, 29, 52] found that vitamin C significantly reduced the incidence of respiratory symptoms originating from the lower respiratory tract, but not the incidence of runny nose [53]. In both RCTs, the proportion of “nose colds” was two-thirds of all colds, and thus the possible effects of vitamin C on symptoms originating from the lower airways are camouflaged if the two types of symptoms are pooled. Thus, vitamin C trials carried out with participants who have only mild forms of a disease may find misleadingly small effects compared with administration of vitamin C to participants who are severely ill. Given the evidence indicating that vitamin C has a greater effect on the more severe forms of the common cold, it is possible that the vitamin may also have an effect on some of the complications of the common cold. Systematic reviews have indicated that vitamin C may be beneficial for common cold-induced asthma [54] and pneumonia [55, 56].

In Fig. 2, we assumed that there was a uniform effect across all included trials. This assumption is simplistic, and it is evident that the effect of vitamin C varies between contexts. Heterogeneity is seen in the Anderson et al. (1972) trial which was sufficiently large to allow informative subgroup analyses on the same outcome over the population (Fig. 4). Possible heterogeneity of the vitamin C effect over sex was also indicated in that the effects appeared to be greater for males [53, 57]. Thus, the 15% and 26% effects of vitamin C on severe common cold outcomes (Figs. 2 and 3) should not be considered as universal estimates, though they are useful in that they reflect an approximation for the magnitude of the effect.

In several of the included trials, control groups were over-exposed with participants receiving high levels of vitamin C either through diet or supplementation. Contamination leads to a smaller observed benefit in the vitamin C group than if it had been compared to a control group receiving only the recommended dose. However, the size of the bias towards the null effect cannot be estimated.

In our Cochrane review we calculated that vitamin C taken regularly in doses ≥ 0.2 g/day shortened colds by 9.4% [19]. That estimate was based on 31 comparisons and should not be directly compared with the current analysis intended to directly compare the effects of vitamin C on mild vs. severe colds. Many outcomes in our Cochrane analysis were mild colds, and many trials used vitamin C doses < 1 g/day [19].

Our analysis was focused on trials in which vitamin C was administered regularly to people in good health. The subsequent severity of cold episodes during the supplementation period was recorded. The evidence of benefit from regular vitamin C supplementation is very strong (Fig. 2). However, if vitamin C alleviates cold symptoms, it seems impractical to take 1 g/day vitamin C regularly over many months to alleviate symptoms from colds which are usually infrequent. Instead, therapeutic vitamin C should be considered. A few therapeutic trials have been carried out, but they have methodological limitations such as initiating treatment too late and not continuing treatment for long enough [19, 27]. Nevertheless, two therapeutic RCTs are relevant when considering the current findings.

In their third trial, Anderson et al. (1975) administered 1.5 g therapeutic vitamin C on the first day, and thereafter 1 g/day for a total of 5 days [58]. Duration of time confined indoors was decreased by 25% (95% CI: -45% to + 1%), whereas mild common cold symptoms were shortened only by 7% (95% CI: -23% to + 13%) (see Additional file 1). This pattern is consistent with that seen in Fig. 3. Karlowski et al. compared 3 g/day regularly with 3 g/day therapeutically for 5 days [25]. There was no indication that the therapeutic administration was less effective; in fact, therapeutic vitamin C appeared to be more effective [23, 24]. Thus, the findings in Fig. 3 encourage investigation of outcomes reflecting different degrees of severity in future therapeutic RCTs on vitamin C and the common cold.

The common cold covers symptoms caused by over a hundred unrelated viruses, the distribution of which varies over time and location. Consequently, virus types have varied between common cold trials and it is unlikely that the benefit of vitamin C is explained by effects on just a certain respiratory virus or virus group. Thus, it seems possible that vitamin C may also have an effect on SARS-CoV-2 infection [59]. A re-analysis of the COVID A to Z trial indicated that therapeutic 8 g/day of vitamin C for 10 days was beneficial [23, 60, 61].

One limitation of our study is that we use the duration of overall symptoms as an approximation for the duration of mild symptoms. The overall duration also includes periods of severe symptoms; however, the durations of severe symptoms are relatively short. Over the 10 study groups in Fig. 3, the median proportion of “mild” symptoms is 77% of the overall duration (Additional file 2), hence the approximation in our analyses does not seem inappropriate. The contexts and outcome definitions of the included trials are heterogeneous enough that we preferred to use the overall duration instead of calculating the duration of mild symptoms by subtracting the severe symptoms from the total duration of symptoms. Furthermore, even if the overall duration of a cold remains constant after vitamin C administration, it is not a bad thing as long as the duration of severe symptoms is reduced. It is the severe symptoms that impact activities of daily life and necessitate taking days off work or school.

Another limitation of our study is that in Fig. 3 we analyze the severe and mild (overall) symptoms as if they were independent, though it is evident that they are correlated as they arise from the same individuals. Therefore, the calculated P-value should be interpreted somewhat cautiously. Nevertheless, this does not change our conclusion that further trials on vitamin C and the common cold should record and analyze cold symptoms so that possible differences in the effects on symptoms of differing severity can be evaluated.

Long-term bias against vitamin C and other vitamins has been documented [18, 23, 62, 63]. Nevertheless, the effect of vitamin C on respiratory infections is a popular topic and several analyses have been published [64–71]. Unfortunately, many of them are erroneous even to the extent that some have been retracted [18, 21–24, 72–82].

In conclusion, we found a significant difference in the effect of vitamin C on mild vs. severe outcomes of the common cold. Vitamin C substantially decreased the severity of colds without influencing their overall duration. Given the low cost and safety of vitamin C, the 15–26% decrease in cold severity may justify regular vitamin C administration in some contexts, such as for people who have frequent contact with young children. Further research should be carried out to estimate the effect of therapeutic vitamin C which is started immediately after the onset of early common cold symptoms. In future studies, various outcome definitions should be considered to enable a more detailed understanding of the effects of vitamin C.

### Electronic supplementary material

Below is the link to the electronic supplementary material.

Supplementary Material 1: Search for the trials, Figs. S1–S5, Description of the included trials.

Supplementary Material 2: Spreadsheet describing the calculations.

## Data Availability

All data generated or analysed during this study are included in this published article and its supplementary information files.
