# Supplementary material for: Vitamin C reduces the severity of common colds: a meta-analysis
Source: BMC Public Health. 2023 Dec 11;23:2468. doi: 10.1186/s12889-023-17229-8 (PMC10712193; doi:10.1186/s12889-023-17229-8)
Supplement: Supplementary file 1 — Supplementary Material 1: Search for the trials, Figs. S1–S5, Description of the included trials. [file 12889_2023_17229_MOESM1_ESM.pdf]

Vitamin C reduces the severity of common colds: a meta-analysis

Harri Hemilä, Elizabeth Chalker

Supplement to a report in:
https://bmcpublichealth.biomedcentral.com

Harri Hemilä, MD, PhD
Department of Public Health,
University of Helsinki, POB 41,
Helsinki, FI-00014, FINLAND.
harri.hemila@helsinki.fi
https://www.mv.helsinki.fi/home/hemila
https://orcid.org/0000-0002-4710-307X

2023-9-19

Contents **Page**

Search for the controlled trials 2

Fig. S1. Exclusion of the three largest trials 4

Fig. S2. Comparison of the effects of vitamin C on severe and mild (overall) common cold symptoms in 15 trial arms 5

Fig. S3. Sensitivity analysis of Fig. 2 using the SMD scale 6

Fig. S4. Sensitivity analysis of Fig. 2 using the random-effects meta-analysis 7

Fig. S5. Effect of vitamin C on confined indoors and any symptoms in Anderson (1975) 8

Calculation of SD for absence from school in the Ludvigsson (1977) trial 9

Table S1. Characteristics of included studies 10

Search for the controlled trials (2023-5-6)

Previously, for our Cochrane review in 2013 we thoroughly searched the literature for vitamin C and common cold trials prior to 2013 [19]. There are also other thorough literature searches up to the 1980s [S1,S2,S3].

For this current analysis we searched for vitamin C and common cold trials published since 2013.

We searched PubMed using search terms [("vitamin C" OR ascorb*) AND "common cold"] with restriction to "clinical trials" and years 2013-2023. We found 3 records, two of which reported clinical trials [34,S4]. Both trials were excluded, see below.

We also searched Scopus using search [TITLE-ABS-KEY("vitamin C" OR ascorb*) AND TITLE-ABS-KEY ("common cold")] limiting to years 2013-2023. We found 180 documents, which included the same two identified with PubMed [34,S4], but no further controlled trials.

We searched the Cochrane CENTRAL Register with search ["vitamin c" or ascorb* in All Text AND "common cold" in All Text]. We found 17 records and the two controlled trials mentioned above [34,S4], but there were no further controlled trials.

Given the possibility that a common cold trial might use key words that are not captured with our search terms above, we also carried out a Web of Science search for the major vitamin C trials.

Our search for papers published since 2013 that had referred to Anderson (1972) [21] or Anderson (1974) [19] found 21 citing articles, but there were no further controlled trials.

Our search for papers published since 2013 that had referred to Pitt (1979) [41] found 17 citing articles, including one of the two mentioned above [S4], but there were no further controlled trials.

Finally, there are a few recent systematic reviews on vitamin C and the common cold [62,63], but they did not refer to any recent trials on vitamin C and the common cold.

Exclusion of the 2 identified trials

The two new trials were by Johnson et al. [34] and Kim et al. [S4].

Johnston et al. [34] excluded mild colds from their recording, describing in their Methods:

> "... Cold symptom severity (10 questions) and the impact of cold symptoms on daily living (9 questions) were assessed daily on a 7-point scale from "very mildly" to "severely". Question scores were summed for each category to calculate the total symptom severity score and the total "impact of cold on daily living" score. For this research, a cold episode was defined as a daily score of 5 or greater for the symptom severity category, indicating either (1) the presence of five different "very mild" cold symptoms; (2) the presence of several different cold symptoms, some mild and some more moderate in severity; or (3) the presence of a single cold symptom at least moderate in severity." (Section 2.3 in [34]).

Given that the purpose of our study was to compare the effect of vitamin C on mild (overall) colds, and on severity outcomes, exclusion of the mild colds in the Johnston et al. [34] trial makes it uninformative for our analyses. In addition, the total number of cold episodes was only 29 with resulting very wide 95%CIs. Consequently, this trial was excluded from our analysis.

Kim et al. [S4] did not report severity outcomes. In addition, the trial had severe flaws which were described elsewhere [S5] and this trial was also excluded from our analysis.

References

19. Hemilä H, Chalker E. Vitamin C for preventing and treating the common cold. Cochrane Database Syst Rev. 2013;2013:CD000980. https://doi.org/10.1002/14651858.cd000980.pub4
https://www.ncbi.nlm.nih.gov/pmc/articles/pmc8078152

26. Anderson TW, Suranyi G, Beaton GH. The effect on winter illness of large doses of vitamin C. Can. Med. Assoc. J. 1974;111:31–36. https://www.ncbi.nlm.nih.gov/pmc/articles/pmc1947567

28. Anderson TW, Reid DB, Beaton GH. Vitamin C and the common cold: A double-blind trial. Can. Med. Assoc. J. 1972;107:503–508. https://www.ncbi.nlm.nih.gov/pmc/articles/pmid/5057006

34. Johnston CS, Barkyoumb GM, Schumacher SS. Vitamin C supplementation slightly improves physical activity levels and reduces cold incidence in men with marginal vitamin C status: a randomized controlled trial. Nutrients. 2014;6(7):2572-83.
https://doi.org/10.3390/nu6072572

41. Pitt HA, Costrini AM. Vitamin C prophylaxis in marine recruits. JAMA 1979;241:908–911.
https://doi.org/10.1001/jama.1979.03290350028016
https://www.researchgate.net/publication/22773196
https://www.academia.edu/78271637

S1. Kleijnen J, ter Riet G, Knipschild PG. Vitamin C and the common cold; review of a megadose of literature (in Dutch) Ned Tijdschr Geneeskd 1989;133:1532–1535.
https://www.ntvg.nl/artikelen/vitamine-c-en-verkoudheid-overzicht-van-een-megadosis-literatuur
https://doi.org/10.5281/zenodo.7965914 (reference list)
https://www.mv.helsinki.fi/home/hemila/CC/Kleijnen_1989.pdf

See problems in the Kleijnen review:
In: Do vitamins C and E affect respiratory infections? Thesis 2006 pp. **38-40**
https://hdl.handle.net/10138/20335
https://doi.org/10.5281/zenodo.6395595

S2. Kleijnen J, Knipschild P. The comprehensiveness of Medline and Embase computer searches. Pharmaceutisch Weekblad Scientific edition 1992;14(5):316–20.
https://doi.org/10.1007/bf01977620

S3. Briggs M. Vitamin C and infectious disease: a review of the literature and the results of a randomised double blind prospective study over eight years. In: Briggs MH editor (s). Recent Vitamin Research. Boca Raton, FL: CRC Press, 1984:39–82.
https://doi.org/10.5281/zenodo.7965806 (reference list)

S4. Kim TK, Lim HR, Byun JS. Vitamin C supplementation reduces the odds of developing a common cold in Republic of Korea Army recruits: randomised controlled trial. BMJ Mil Health. 2022;168(2):117-123.
https://doi.org/10.1136/bmjmilitary-2019-001384

S5. Hemilä H. Statistical problems in the vitamin C and common cold trial with South Korean army recruits [Rapid response 2020-4-14].
https://militaryhealth.bmj.com/content/168/2/117.responses

Exclusion of the three largest trials

In Figure S1 below, the three largest trials by Anderson (1972), Ludvigsson (1977L) and Pitt (1979) are excluded from the analysis shown in Figure 2 of the report, yet there remains a significant 19% effect on the severe common cold outcomes.

Fig. S1. Exclusion of the three largest trials

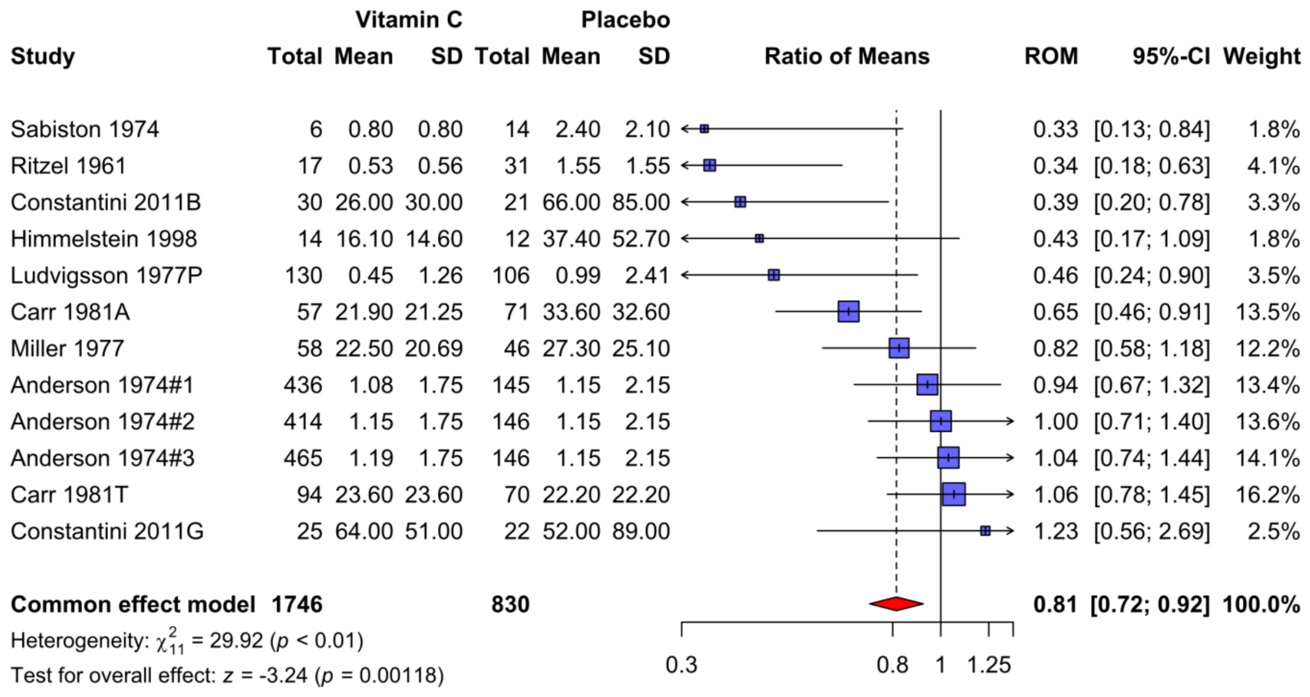

Fig. S2. Comparison of the effects of vitamin C on severe and mild (overall) common cold symptoms in 15 trial arms

Study	Vitamin C Total	Vitamin C Mean	Vitamin C SD	Placebo Total	Placebo Mean	Placebo SD	ROM	95%-CI
**Severe Outcome**								
Sabiston 1974	6	0.80	0.80	14	2.40	2.10	0.33	[0.13; 0.84]
Ritzel 1961	17	0.53	0.56	31	1.55	1.55	0.34	[0.18; 0.63]
Constantini 2011B	30	26.00	30.00	21	66.00	85.00	0.39	[0.20; 0.78]
Himmelstein 1998	14	16.10	14.60	12	37.40	52.70	0.43	[0.17; 1.09]
Ludvigsson 1977P	130	0.45	1.26	106	0.99	2.41	0.46	[0.24; 0.90]
Carr 1981A	57	21.90	21.25	71	33.60	32.60	0.65	[0.46; 0.91]
Anderson 1972	561	1.04	1.75	609	1.32	2.15	0.79	[0.65; 0.95]
Ludvigsson 1977L	657	0.95	1.66	622	1.16	2.10	0.82	[0.67; 0.99]
Miller 1977	58	22.50	20.69	46	27.30	25.10	0.82	[0.58; 1.18]
Pitt 1979	600	0.87	0.76	619	0.97	0.84	0.90	[0.81; 0.99]
Anderson 1974#1	436	1.08	1.75	145	1.15	2.15	0.94	[0.67; 1.32]
Anderson 1974#2	414	1.15	1.75	146	1.15	2.15	1.00	[0.71; 1.40]
Anderson 1974#3	465	1.19	1.75	146	1.15	2.15	1.04	[0.74; 1.44]
Carr 1981T	94	23.60	23.60	70	22.20	22.20	1.06	[0.78; 1.45]
Constantini 2011G	25	64.00	51.00	22	52.00	89.00	1.23	[0.56; 2.69]
**Common effect model**	**3564**			**2680**			**0.85**	**[0.79; 0.91]**
Test for effect in subgroup: z = -4.8 (p = 0.000002)								
**Total duration**								
Constantini 2011B	30	5.50	5.00	21	10.40	7.10	0.53	[0.34; 0.82]
Himmelstein 1998	14	2.50	1.10	12	4.20	3.50	0.60	[0.35; 1.01]
Carr 1981A	57	4.86	3.56	71	7.50	5.50	0.65	[0.50; 0.84]
Ritzel 1961	17	1.80	1.01	31	2.60	1.46	0.69	[0.50; 0.96]
Ludvigsson 1977P	130	5.39	4.88	106	7.61	8.07	0.71	[0.55; 0.91]
Sabiston 1974	6	4.20	3.80	14	5.60	2.80	0.75	[0.35; 1.62]
Anderson 1974#3	465	3.23	3.84	146	3.52	3.43	0.92	[0.76; 1.11]
Miller 1977	58	7.70	4.27	46	8.30	4.60	0.93	[0.75; 1.15]
Anderson 1972	561	3.96	3.84	609	4.18	3.43	0.95	[0.85; 1.05]
Anderson 1974#2	414	3.35	3.84	146	3.52	3.43	0.95	[0.78; 1.15]
Anderson 1974#1	436	3.42	3.84	145	3.52	3.43	0.97	[0.80; 1.17]
Pitt 1979	600	11.20	11.20	619	11.50	11.50	0.97	[0.87; 1.09]
Carr 1981T	94	5.46	5.46	70	5.42	5.42	1.01	[0.74; 1.37]
Ludvigsson 1977L	657	6.04	5.47	622	5.67	7.89	1.07	[0.94; 1.21]
Constantini 2011G	25	8.60	5.50	22	7.40	8.20	1.16	[0.69; 1.97]
**Common effect model**	**3564**			**2680**			**0.93**	**[0.88; 0.98]**
Test for effect in subgroup: z = -2.9 (p = 0.003304)								

Test for subgroup differences: χ^2_1 = 4.35, df = 1 (p = 0.037)

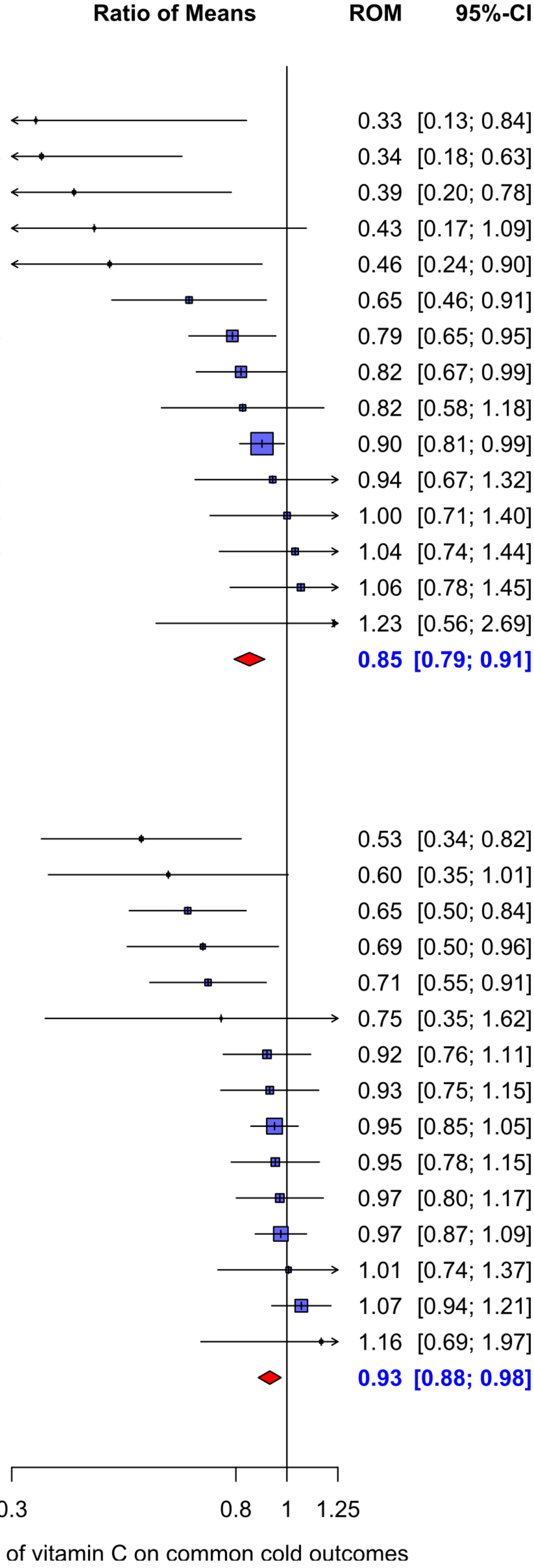

Fig. S3. Sensitivity analysis of Fig. 2 using the SMD scale

Study	Vitamin C Total	Vitamin C Mean	Vitamin C SD	Placebo Total	Placebo Mean	Placebo SD	SMD	95%-CI	Weight
**Days confined to house or off school**									
Ludvigsson 1977P	130	0.45	1.26	106	0.99	2.41	-0.28	[-0.54; -0.03]	4.0%
Anderson 1972	561	1.04	1.75	609	1.32	2.15	-0.14	[-0.26; -0.03]	20.2%
Ludvigsson 1977L	657	0.95	1.66	622	1.16	2.10	-0.11	[-0.22; -0.00]	22.2%
Anderson 1974#1	436	1.08	1.75	145	1.15	2.15	-0.04	[-0.22; 0.15]	7.6%
Anderson 1974#2	414	1.15	1.75	146	1.15	2.15	0.00	[-0.19; 0.19]	7.5%
Anderson 1974#3	465	1.19	1.75	146	1.15	2.15	0.02	[-0.16; 0.21]	7.7%
**Common effect model**	**2663**			**1774**			**-0.10**	**[-0.16; -0.03]**	**69.2%**
Heterogeneity: $\chi^2_5 = 5.69$ (p = 3.4e-01)									
Test for effect in subgroup: z = -3.01 (p = 2.6e-03)									
**Days of severe symptoms**									
Sabiston 1974	6	0.80	0.80	14	2.40	2.10	-0.84	[-1.83; 0.16]	0.3%
Ritzel 1961	17	0.53	0.56	31	1.55	1.55	-0.78	[-1.39; -0.16]	0.7%
**Common effect model**	**23**			**45**			**-0.79**	**[-1.31; -0.27]**	**1.0%**
Heterogeneity: $\chi^2_1 = 0.01$ (p = 9.2e-01)									
Test for effect in subgroup: z = -2.97 (p = 3.0e-03)									
**Severity scale**									
Constantini 2011B	30	26.00	30.00	21	66.00	85.00	-0.67	[-1.24; -0.09]	0.8%
Himmelstein 1998	14	16.10	14.60	12	37.40	52.70	-0.55	[-1.34; 0.23]	0.4%
Carr 1981A	57	21.90	21.25	71	33.60	32.60	-0.41	[-0.77; -0.06]	2.1%
Miller 1977	58	22.50	20.69	46	27.30	25.10	-0.21	[-0.60; 0.18]	1.8%
Pitt 1979	600	0.87	0.76	619	0.97	0.84	-0.12	[-0.24; -0.01]	21.1%
Carr 1981T	94	23.60	23.60	70	22.20	22.20	0.06	[-0.25; 0.37]	2.8%
Constantini 2011G	25	64.00	51.00	22	52.00	89.00	0.17	[-0.41; 0.74]	0.8%
**Common effect model**	**878**			**861**			**-0.15**	**[-0.24; -0.05]**	**29.9%**
Heterogeneity: $\chi^2_6 = 9.5$ (p = 1.5e-01)									
Test for effect in subgroup: z = -3.03 (p = 2.5e-03)									
**Common effect model**	**3564**			**2680**			**-0.12**	**[-0.17; -0.07]**	**100.0%**
Heterogeneity: $\chi^2_{14} = 22.44$ (p = 7.0e-02)									
Test for overall effect: z = -4.45 (p = 8.6e-06)									
Test for subgroup differences: $\chi^2_2 = 7.24$, df = 2 (p = 2.7e-02)									

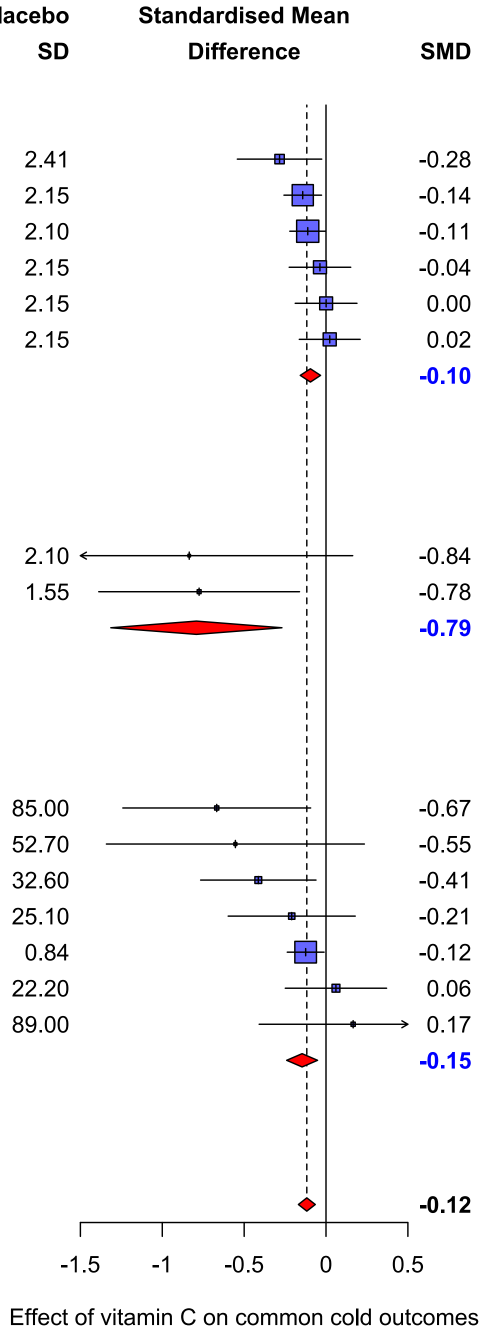

Fig. S4. Sensitivity analysis of Fig. 2 using the random-effects meta-analysis

Study	Vitamin C Total	Vitamin C Mean	Vitamin C SD	Placebo Total	Placebo Mean	Placebo SD	ROM	95%-CI	Weight
**Days confined to house or off school**									
Ludvigsson 1977P	130	0.45	1.26	106	0.99	2.41	0.46	[0.24; 0.90]	3.2%
Anderson 1972	561	1.04	1.75	609	1.32	2.15	0.79	[0.65; 0.95]	11.6%
Ludvigsson 1977L	657	0.95	1.66	622	1.16	2.10	0.82	[0.67; 0.99]	11.4%
Anderson 1974#1	436	1.08	1.75	145	1.15	2.15	0.94	[0.67; 1.32]	7.7%
Anderson 1974#2	414	1.15	1.75	146	1.15	2.15	1.00	[0.71; 1.40]	7.8%
Anderson 1974#3	465	1.19	1.75	146	1.15	2.15	1.04	[0.74; 1.44]	7.9%
**Random effects model**	**2663**			**1774**			**0.85**	**[0.76; 0.94]**	**49.6%**
Heterogeneity: $\chi^2_5 = 6.61$ (p = 2.5e-01)									
Test for effect in subgroup: z = -2.97 (p = 3.0e-03)									
**Days of severe symptoms**									
Sabiston 1974	6	0.80	0.80	14	2.40	2.10	0.33	[0.13; 0.84]	1.9%
Ritzel 1961	17	0.53	0.56	31	1.55	1.55	0.34	[0.18; 0.63]	3.7%
**Random effects model**	**23**			**45**			**0.34**	**[0.20; 0.57]**	**5.6%**
Heterogeneity: $\chi^2_1 = 0$ (p = 9.6e-01)									
Test for effect in subgroup: z = -4.14 (p = 3.4e-05)									
**Severity scale**									
Constantini 2011B	30	26.00	30.00	21	66.00	85.00	0.39	[0.20; 0.78]	3.1%
Himmelstein 1998	14	16.10	14.60	12	37.40	52.70	0.43	[0.17; 1.09]	1.9%
Carr 1981A	57	21.90	21.25	71	33.60	32.60	0.65	[0.46; 0.91]	7.7%
Miller 1977	58	22.50	20.69	46	27.30	25.10	0.82	[0.58; 1.18]	7.4%
Pitt 1979	600	0.87	0.76	619	0.97	0.84	0.90	[0.81; 0.99]	13.9%
Carr 1981T	94	23.60	23.60	70	22.20	22.20	1.06	[0.78; 1.45]	8.4%
Constantini 2011G	25	64.00	51.00	22	52.00	89.00	1.23	[0.56; 2.69]	2.5%
**Random effects model**	**878**			**861**			**0.80**	**[0.65; 0.99]**	**44.9%**
Heterogeneity: $\chi^2_6 = 12.92$ (p = 4.4e-02)									
Test for effect in subgroup: z = -2.06 (p = 4.0e-02)									
**Random effects model**	**3564**			**2680**			**0.79**	**[0.69; 0.91]**	**100.0%**
Heterogeneity: $\chi^2_{14} = 32.30$ (p = 3.6e-03)									
Test for overall effect: z = -3.38 (p = 7.2e-04)									
Test for subgroup differences: $\chi^2_2 = 11.74$, df = 2 (p = 2.8e-03)									

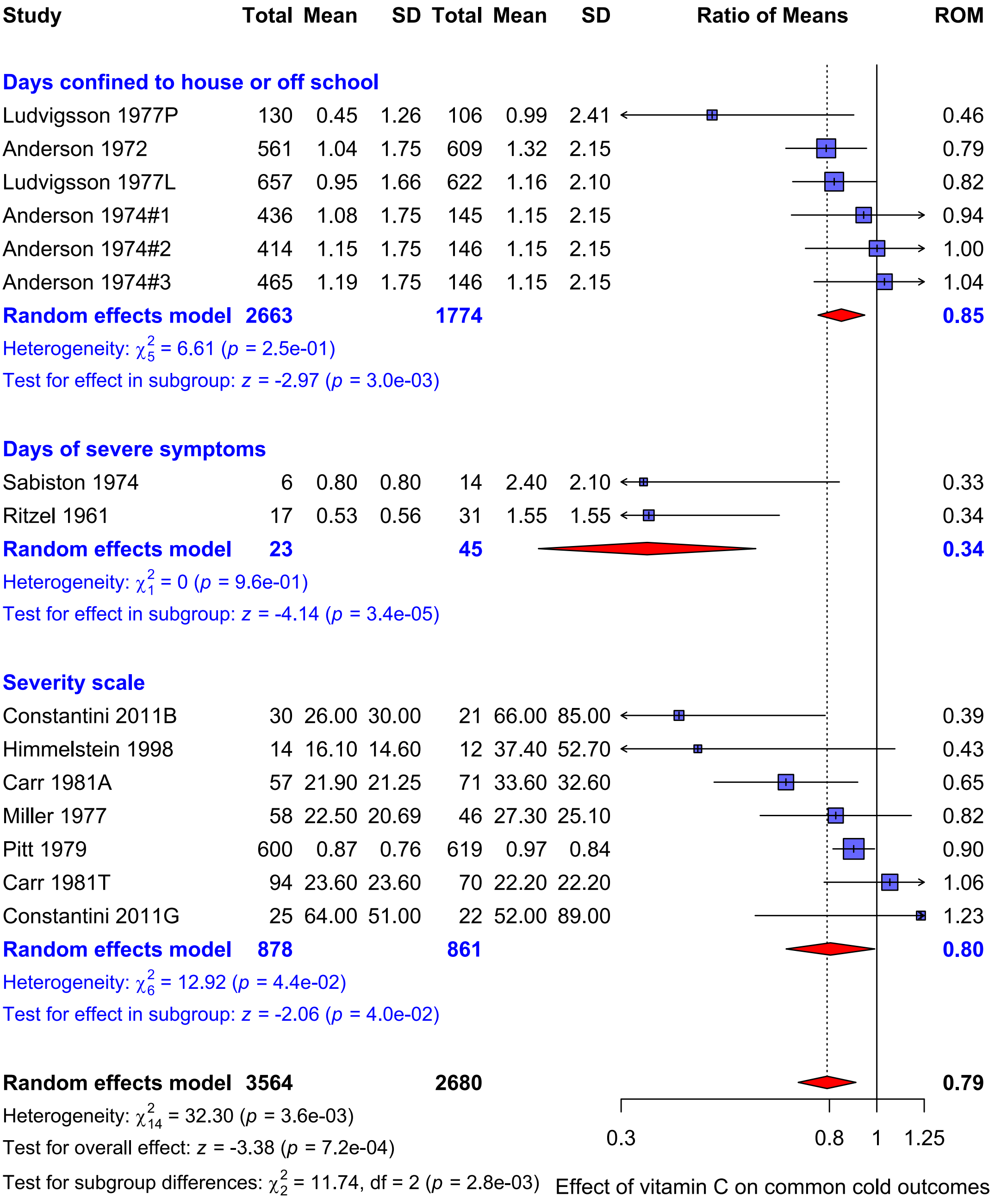

Fig. S5. Effect of vitamin C on any symptoms and days confined indoors in Anderson (1975)

Study	Vitamin C Total	Vitamin C Mean	Vitamin C SD	Placebo Total	Placebo Mean	Placebo SD	ROM	95%-CI
**Any symptoms**								
Vitamin C capsule	152	4.97	6.23	73	5.38	5.09	0.92	[0.69; 1.24]
Vitamin C tablet	150	5.05	4.67	73	5.38	5.09	0.94	[0.72; 1.22]
**Common effect model**	**302**			**146**			**0.93**	**[0.77; 1.13]**
Test for effect in subgroup: $z = -0.7$ ($p = 0.477$)								
**Confined indoors**								
Vitamin C capsule	152	1.22	1.82	73	1.61	2.46	0.76	[0.49; 1.16]
Vitamin C tablet	150	1.19	1.74	73	1.61	2.46	0.74	[0.48; 1.12]
**Common effect model**	**302**			**146**			**0.75**	**[0.55; 1.01]**
Test for effect in subgroup: $z = -1.9$ ($p = 0.056$)								

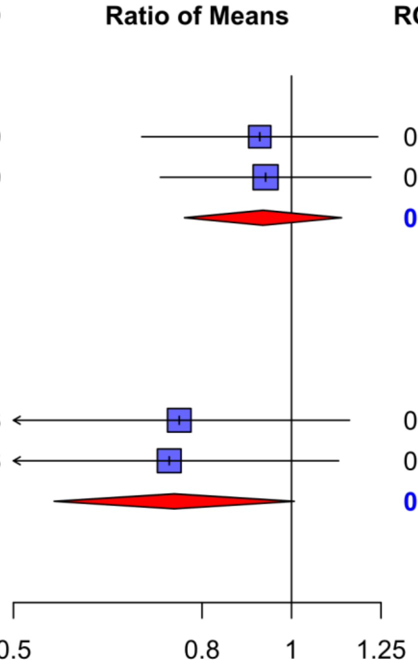

See data in Additional file 2 and in the Anderson et al (1975) report [58].

Calculation of SD for absence from school in the Ludvigsson (1977) trial

See calculations on sheet "Ludvigsson" and data on Table V in Ludvigsson et al. (1977) [30]

From Table V: Absence from school, vitamin C 1000 mg/day group:
Total number of common cold episodes: 1.63*80 = 130
Number of common cold episodes with Absence from school: 0.28*80 = 22.
Number of (mild) common cold episodes without Absence from school: 108 = 130 – 22.
Mean and SD for Absence from school among the 22 is reported in Table V.

Let us assume that there are 130 observations distributed between two groups of
108 observations all with value 0 (mean 0 and SD 0)
22 observations with mean 2.68 and SD 1.88 (Table V in [30]).
The SD for all the 130 observations can be calculated as follows:

The whole group has sum of squares over 130 with $\ddot{X}$ being the overall mean:

$SS = \Sigma_{130}(X_i - \ddot{X})^2$

Variance = SS/129

We can divide the SS into two parts: 22 and 108:

$SS = \Sigma_{22}(X_i - \dot{X}_{22} + \dot{X}_{22} - \ddot{X})^2 + \Sigma_{108}(X_i - \ddot{X})^2$

Here $\dot{X}_{22} = 2.68$ for the subgroup of the 22; and $X_i = 0$ for all the 108

$SS = \Sigma_{22}[(X_i - \dot{X}_{22}) + (\dot{X}_{22} - \ddot{X})]^2 + \Sigma_{108}(\ddot{X})^2$

$SS = \Sigma_{22}[(X_i - \dot{X}_{22})^2 + 2*(X_i - \dot{X}_{22})*(\dot{X}_{22} - \ddot{X}) + (\dot{X}_{22} - \ddot{X})^2] + \Sigma_{108}(\ddot{X})^2$

The term $(X_i - \dot{X}_{22})$ sums to 0, since $\dot{X}_{22}$ is the mean of X_i in the group of the 22.

Thus,

$SS = \Sigma_{22}(X_i - \dot{X}_{22})^2 + \Sigma_{22}(\dot{X}_{22} - \ddot{X})^2 + \Sigma_{108}(\ddot{X})^2$

$\Sigma_{22}[(X_i - \dot{X}_{22})^2 = 21 * Var_{22}$

and

$Var_{22} = SD_{22}$^2 = 1.88^2 =3.5344

Then we need $\ddot{X}$:

$\ddot{X}$ = 22* 2.68/130 = 0.4535

Thus,

$SS = \Sigma_{22}(X_i - \dot{X}_{22})^2 + \Sigma_{22}(\dot{X}_{22} - \ddot{X})^2 + \Sigma_{108}(\ddot{X})^2$

$= 21*3.5344 + 22*(2.68 - 0.4535)^2 +$ 108*0.4535^2

= 74.2 + 109.0 + 22.2 = 205.49

Var = 205.49/129 = 1.593

$SD_{130} = \sqrt{Var} = 1.262$

Table S1. Characteristics of included studies

Anderson 1972

**Methods**	Double-blind RCT. Duration 3 months (Dec 1971 to Mar 1972).<br>https://www.ncbi.nlm.nih.gov/pmc/articles/PMC1940935<br>https://www.ncbi.nlm.nih.gov/pmc/articles/PMC1941144 (Correction 1)<br>https://www.ncbi.nlm.nih.gov/pmc/articles/PMC1941189 (Correction 2)<br>Pauling's summaries:<br>https://scarc.library.oregonstate.edu/coll/pauling/rnb/18/18-099.html<br>https://scarc.library.oregonstate.edu/coll/pauling/rnb/18/18-100.html<br>https://scarc.library.oregonstate.edu/coll/pauling/rnb/18/18-101.html
**Participants**	Canadian adults from a variety of occupations and age groups.<br>"we asked persons not to enrol unless they normally experienced at least one cold in the period January to March, the study group was not representative of the general population in terms of susceptibility to colds" (p 504).<br>Age mean 29 y (10 to 65 y).<br>Males 257, females 461 (p 504).<br>Vitamin C 407; placebo 411.<br>"Subjects who were in the habit of taking a daily multivitamin supplement ... accepted into the study only if the daily dose of vitamin C was 50 mg or less" (p 504).
**Interventions**	1 g/d vitamin C regularly for 3 months and 3 g/d vitamin C extra for the first 3 days of illness.<br>Thus, the dose on the first 3 days of a disease was 4 g/d.<br>"Particular care was taken to ensure that the vitamin and placebo tablets were indistinguishable in appearance and taste. Pure ascorbic acid has a very strong and characteristic flavour which is difficult to imitate, and we therefore used a formulation containing 200 mg of sodium ascorbate, 75 mg of ascorbic acid and an artificial orange flavouring. The taste of this formulation was well matched by a placebo preparation containing 30 mg of citric acid and the same orange flavouring and fillers. The effectiveness of the matching was established by asking 30 individuals to taste both tablets, and using pure ascorbic acid as reference, to judge which tablet contained the vitamin. Sixteen persons selected the placebo tablet and 14 the vitamin tablet. The effectiveness of the matching was verified at the end of the main study by the answers to the question "Do you think you have been on the vitamin or placebo tablet?". Approximately half of the 818 subjects answered "Don't know", and the remainder were divided almost equally between those who guessed correctly and those who did not" (p 504).
**Outcomes**	**Total duration of cold symptoms:**<br>Duration of all colds "symptoms present" (p 505; Table II).<br>**Common cold severity outcome:**<br>Duration of "confined to house" (p 505; Table II).
**Notes**	Funding: not reported

Risk of bias table

Bias	Authors' judgement	Support for judgement
Random sequence generation (selection bias)	Low risk	"Subjects were allocated to vitamin and placebo in a strictly double-blind randomized manner" (p 504).
Allocation concealment (selection bias)	Low risk	See above.
Baseline balance	Low risk	Table 1 shows balance for age, sex, student status, smokers, cold frequency, contact with young children, frequency in crowds, consumption of fruit juices.
Blinding of participants and personnel (performance bias)	Low risk	See above and "... the code was not broken until after all the data had been transferred to punch cards and initial tabulations carried out" (p 504).
Blinding of outcome assessment (detection bias)	Low risk	See above and "[blinded] Subjects were instructed to record each day whether they were sick or well" (p 504-5),
Incomplete outcome data (attrition bias)	Low risk	182 drop outs out of initial full complement of 1000.<br>Almost all were contacted and most dropped out because of loss of interest or inability to remember to take tablets. (p 504) It is not clear whether the drop outs were evenly distributed between the vitamin C and placebo groups, but the relative distribution of recorded

		characteristics was the same as the main group (p 505). There were 28 dropouts "because of suspected side effects, distributed almost equally between the vitamin (15) and placebo groups (13)" (p 507).
Selective reporting (reporting bias)	Low risk	Relevant outcomes were reported extensively. Incidence of colds, duration of "symptoms present", duration of "confined to house", "days off work" and proportions without symptoms, without confined to house, and without days off work.
Vitamin C and placebo indistinguishable?	Low risk	See above: "The taste of this [vitamin C tablet] formulation was well matched by a placebo preparation...The effectiveness of the matching was established by asking 30 individuals to taste both tablets ..." (p 504).
Contamination	Unclear risk	There was insufficient reporting to enable assessment. However, the effect of vitamin C was greater on participants who consumed less fruit juice compared with those who consumed more, which is consistent with baseline vitamin C intake modifying the effects of supplementation.

Anderson 1974#1 Arm #1

**Methods**	Double-blind RCT. Duration 3 months (Dec 1972 to Feb 1973).<br>8 trial arms: 4 regular vitamin C supplementation arms, 2 vitamin C treatment arms, and 2 placebo arms.<br>This entry is the trial **arm #1**: regular 1 g/day + therapeutic 3 g/d supplementation.<br>The other regular vitamin C arms are included as Anderson 1974#2 and 1974#3.<br>https://www.ncbi.nlm.nih.gov/pmc/articles/PMC1947567<br>Comparison of the placebo groups #4 vs #6 (Hemilä 2006: p 40, Table 16):<br>https://hdl.handle.net/10138/20335<br>https://doi.org/10.5281/zenodo.6395595<br>Pauling's summaries:<br>https://scarc.library.oregonstate.edu/coll/pauling/rnb/18/18-121.html<br>https://scarc.library.oregonstate.edu/coll/pauling/rnb/18/18-122.html
**Participants**	Canadian adults, both sexes, recruited from staff of large hospitals and business organisations (p 32).<br>"Although subjects were recruited from a variety of occupations, it should be stressed that they were not a representative sample of the general population, since only those persons who usually suffered at least one episode<br>of illness between December and March (but were otherwise in good general health) were accepted" (p 32).<br>Mean age 34.<br>Males 48%.<br>Vitamin C 277; placebo (arm #4) 285; to avoid triple counting, one third of the placebo group (=285/3) was used in each vitamin C comparison.
**Interventions**	1 g/d vitamin C regularly for 3 months and 3 g/d vitamin C extra at onset of illness on the 1st day only.<br>Thus, the dose on the 1st day of a disease was 4 g/d.<br>"instructed to take four of their "Daily" tablets each day (one qid or two bid) plus 12 of their Extra tablets [0.25 g] (two every hour) on the first day of any illness" (p 32).<br>"All three tablets were of a similar size and shape, and an initial 'taste test' carried out with the help of a number of colleagues demonstrated that they were reasonably well matched in flavour, texture and appearance. This was confirmed at the end of the trial by asking the participants whether they thought their daily tablets had contained vitamin or placebo. Approximately half of each group answered "don't know", and of the remainder, approximately two thirds answered "vitamin" and one third "placebo", irrespective of the actual nature of their tablets" (p 32).
**Outcomes**	**Total duration of cold symptoms:**<br>Duration of all colds "days of symptoms" (p 33; Table II).<br>**Common cold severity outcome:**<br>"Days indoors" (p 33; Table II).
**Notes**	There were problems with the placebo group #6 so that baseline was inconsistent with the baselines of six vitamin C groups (Hemilä 2006: p 40, Table 16):<br>https://hdl.handle.net/10138/20335<br>https://doi.org/10.5281/zenodo.6395595<br>Therefore, comparison in this review is restricted to the placebo group #4 which had close baseline values for "usual days indoors" and "usual days off work" and "contact with children" consistent with the baseline values in the six vitamin C groups.<br>"A labelling error had occurred in two of the 176 batches" (p 33), but the authors argued that this error was taken into account.<br>SD for duration and days indoors was not published and it was imputed from the Anderson 1972 trial, see Additional file.<br>Funding: Hoffmann-La Roche Ltd. supplied the tablets.

Risk of bias table

Bias	Authors' judgement	Support for judgement
Random sequence generation (selection bias)	Low risk	"Bottles were numbered in accordance with a computer-generated list of numbers randomized in groups of eight, then given out in consecutive order as subjects registered" (p 33).
Allocation concealment (selection bias)	Low risk	"double-blind" (p 31).

Baseline balance	Low risk	Compared with the placebo group #4, Table 2 shows balance for age, sex, smoking, frequency of usual cold episodes, usual days indoors, usual days off work, contact with children, frequency in crowds, daily juice consumption.
Blinding of participants and personnel (performance bias)	Low risk	"double-blind" (p 31).
Blinding of outcome assessment (detection bias)	Low risk	Blinded "subjects were asked to complete a checklist of the symptoms present on each day of illness" (p 33).
Incomplete outcome data (attrition bias)	Low risk	The dropout rates were 37% (163/440) in the vitamin C group and 35% (155/440) in the placebo group #4. Of the total 1171 subjects who dropped out of the study, 74 cited side effects as the reason (p 35). One of the commonest reasons for dropping out was difficulty in swallowing the large tablets (p 34).
Selective reporting (reporting bias)	Low risk	Relevant outcomes were reported extensively.<br>Incidence of colds, duration of "symptoms present", "days indoors", "days off work".
Vitamin C and placebo indistinguishable?	Low risk	"All three tablets were of a similar size and shape, and an initial 'taste test' carried out with the help of a number of colleagues demonstrated that they were reasonably well matched in flavour, texture and appearance" (p 32).
Contamination	Unclear risk	There was insufficient reporting to enable assessment.

Anderson 1974#2 Arm #2

**Methods**	See Anderson 1974#1<br>This entry is the trial **arm #2**: regular 1 g/d vit C supplementation.
**Participants**	See Anderson 1974#1<br>Vitamin C 275; placebo (arm #4) 285; to avoid triple counting, one third of the placebo group (=285/3) was used in each vitamin C comparison.
**Interventions**	1 g/d vitamin C regularly for 3 months<br>See Anderson 1974#1
**Outcomes**	See Anderson 1974#1
**Notes**	See Anderson 1974#1

Risk of bias table

Bias	Authors' judgement	Support for judgement
Random sequence generation (selection bias)	Low risk	See Anderson 1974#1
Allocation concealment (selection bias)	Low risk	See Anderson 1974#1
Baseline balance	Low risk	See Anderson 1974#1
Blinding of participants and personnel (performance bias)	Low risk	See Anderson 1974#1
Blinding of outcome assessment (detection bias)	Low risk	See Anderson 1974#1
Incomplete outcome data (attrition bias)	Low risk	See Anderson 1974#1
Selective reporting (reporting bias)	Low risk	See Anderson 1974#1
Vitamin C and placebo indistinguishable?	Low risk	See Anderson 1974#1
Contamination	Unclear risk	See Anderson 1974#1

Anderson 1974#3 Arm #3

**Methods**	See Anderson 1974#1<br>This entry is the trial **arm #3**: regular 2 g/d vit C supplementation.
**Participants**	See Anderson 1974#1<br>Vitamin C 308; placebo (arm #4) 285; to avoid triple counting, one third of the placebo group (=285/3) was used in each vitamin C comparison.
**Interventions**	2 g/d vitamin C regularly for 3 months<br>See Anderson 1974#1
**Outcomes**	See Anderson 1974#1
**Notes**	See Anderson 1974#1

Risk of bias table

Bias	Authors' judgement	Support for judgement
Random sequence generation (selection bias)	Low risk	See Anderson 1974#1
Allocation concealment (selection bias)	Low risk	See Anderson 1974#1
Baseline balance	Low risk	See Anderson 1974#1
Blinding of participants and personnel (performance bias)	Low risk	See Anderson 1974#1
Blinding of outcome assessment (detection bias)	Low risk	See Anderson 1974#1
Incomplete outcome data (attrition bias)	Low risk	See Anderson 1974#1
Selective reporting (reporting bias)	Low risk	See Anderson 1974#1
Vitamin C and placebo indistinguishable?	Low risk	See Anderson 1974#1
Contamination	Unclear risk	See Anderson 1974#1

Carr 1981A living Apart

**Methods**	Double-blind RCT. Duration 100 days, beginning Jun 1980 (including the worst of the winter weather).<br>Identical twins: 1 group living together and 1 group living apart.<br>This comparison includes twins living **apart**.<br>https://doi.org/10.1017/s0001566000006450 (Acta Genet Med Gemellol 1981)<br>https://genepi.qimr.edu.au/contents/p/staff/CV020.pdf<br>https://doi.org/10.5694/j.1326-5377.1981.tb101032.x (Med J Aust 1981)<br>https://genepi.qimr.edu.au/contents/p/staff/CV019.pdf<br>https://www.researchgate.net/publication/16306809 (Human Genetics 1982)
**Participants**	Australian males and females.<br>Age mean 25 y (range 14 to 64 y; 36 pairs under 18 years, 34 pairs aged 18 to 30 years, and 25 pairs aged 30+ years).<br>38 male and 57 female pairs of twins in total.<br>This comparison is for 44 twin pairs living **apart**.
**Interventions**	1 g/d vitamin C for 100 days.<br>"The treatment group received 1 g of ascorbic acid per day in the form of Redoxon® tablets (Roche Products),<br>and the control group received a placebo with the same ingredients in different proportions but with lactose substituted for ascorbic acid. Quality of matching of the active and placebo tablets was checked for both appearance and taste by triangular discrimination tests on 60 pharmacology students. Two of one kind of tablet and one of the other were presented to the subject who had to pick the odd one out. It was found that subjects could pick a difference between the appearance of the dry tablets (chi2 = 39.7) but when these were dissolved in water they were not able to pick a difference at better than chance level (chi2 = 0.68). Having decided on the solution that tasted different, subjects were asked to identify it as active or placebo and in fact fewer than expected by chance were able to identify the solution correctly. It was concluded that the placebo was well matched in important respects and subsequent results bore this out" (p 250).<br>"In addition ..., both groups received multivitamin capsules containing 70 mg vitamin C. This was to ensure that any observed treatment effect could reasonably be attributed to the pharmacologic dose of vitamin C and not to alleviation of dietary deficiency" (p 250).
**Outcomes**	**Total duration of cold symptoms:**<br>Common cold duration (p 252; Table 2).<br>**Common cold severity outcome:**<br>Common cold severity (p 252; Table 2).
**Notes**	The SD for duration and were not published and the SDs were calculated from the P values, setting the ratio of SD in the vitamin C and placebo groups equal with the ratio of mean outcomes in the vitamin C and placebo groups, see Additional file.<br>"Among the twins living together, those taking vitamin C had a significantly higher incidence, total duration, and total severity of colds ... Among the pairs living apart there were 9 significant treatment differences ... all of these favoured the vitamin C group" (p 252).<br>Funding: Roche Products, supplied the tablets and gave financial support to cover postage costs.

Risk of bias table

Bias	Authors' judgement	Support for judgement
Random sequence generation (selection bias)	Low risk	"One twin of a pair was assigned at random to the treatment groups and the other to the control group" (p 250).
Allocation concealment (selection bias)	Low risk	"The experiment was "double-blind" in that neither the subjects nor the experimenters involved with the subjects or with the analysis of the results knew which group was which until the experiment and the analysis were completed" (p 250).
Baseline balance	Low risk	Twins
Blinding of participants and personnel (performance bias)	Low risk	See above.
Blinding of outcome assessment (detection bias)	Low risk	See above.
Incomplete outcome data (attrition bias)	Low risk	"Of the 125 pairs of twins who began the trial, we have analyzed cold data for 95 pairs" (p 250)

		Because of the pairing, the dropout of a pair cannot generate a systematic bias between the groups.
Selective reporting (reporting bias)	Low risk	Incidence of colds, duration of colds and severity of colds reported.
Vitamin C and placebo indistinguishable?	Low risk	"matching of the active and placebo tablets was checked for both appearance and taste" (p 250).
Contamination	High risk	Placebo group received a multi-vitamin tablet containing 70 mg/d vitamin C. In addition, no effect of vitamin C was seen among twins who lived together, whereas a significant benefit of vitamin C was seen among twins living apart, which most probably is explained by swapping of tablets among twins living together.

Carr 1981T living Together

**Methods**	Double-blind RCT. Duration 100 days, beginning Jun 1980 (including the worst of the winter weather).<br>Identical twins: 1 group living together and 1 group living apart.<br>This comparison includes twins living **together**.<br>See Carr 1981A.
**Participants**	Australian males and females.<br>Age mean 25 y (range 14 to 64 y; 36 pairs under 18 years, 34 pairs aged 18 to 30 years, and 25 pairs aged 30+ years).<br>38 male and 57 female pairs of twins in total.<br>This comparison is for 51 twin pairs living **together**.
**Interventions**	See Carr 1981A
**Outcomes**	See Carr 1981A
**Notes**	See Carr 1981A<br>SD was not reported. For this comparison of twins living together, we imputed SD = mean.

Risk of bias table

Bias	Authors' judgement	Support for judgement
Random sequence generation (selection bias)	Low risk	See Carr 1981A
Allocation concealment (selection bias)	Low risk	See Carr 1981A
Baseline balance	Low risk	See Carr 1981A
Blinding of participants and personnel (performance bias)	Low risk	See Carr 1981A
Blinding of outcome assessment (detection bias)	Low risk	See Carr 1981A
Incomplete outcome data (attrition bias)	Low risk	See Carr 1981A
Selective reporting (reporting bias)	Low risk	See Carr 1981A
Vitamin C and placebo indistinguishable?	Low risk	See Carr 1981A
Contamination	High risk	Placebo group received a multi-vitamin tablet containing 70 mg/d vitamin C. In addition, no effect of vitamin C was seen among twins who lived together, whereas a significant benefit of vitamin C was seen among twins living apart, which most probably is explained by swapping of tablets among twins living together (See Carr 1981A).

Constantini 2011F Females

**Methods**	Double-blind RCT. Duration 3 winter months.<br>https://doi.org/10.1007/s00431-010-1270-z<br>https://www.researchgate.net/publication/45509345<br>https://helda.helsinki.fi/handle/10138/144198<br>This comparison is **female** swimmers
**Participants**	Israel. **Female** competitive swimmers with a training volume of at least 15 h/week.<br>Mean age over both sexes 14 y (12 to 17 y).<br>"Exclusion criteria were the presence of chronic health conditions and the use of prescription medications or dietary supplementations of any kind" (p 60).<br>Vitamin C 9; placebo 8.
**Interventions**	1 g/d vitamin C for 3 months.<br>"The vitamin (ascorbic acid) and placebo pills were prepared by a pharmaceutical company ... and were identical in appearance" (p 60).
**Outcomes**	**Total duration of cold symptoms:**<br>Common cold duration (p 62; Table 3).<br>**Common cold severity outcome:**<br>Common cold severity (p 62; Table 3).
**Notes**	The trial is divided into male and female comparisons since there was a significant heterogeneity in vitamin C effect (P = 0.003).<br>The tablets for the study were provided by Teva Pharmaceutical Industries Ltd, Israel.

Risk of bias table

Bias	Authors' judgement	Support for judgement
Random sequence generation (selection bias)	Low risk	"before their distribution to the swimmers, [plastic bottles (identical number of vitamin C and placebo bottles)] were numbered by a person unrelated to the study. Study participants were given a randomly selected plastic bottle, and its number was listed alongside the participant's name" (p 60).
Allocation concealment (selection bias)	Low risk	See above.
Baseline balance	Low risk	Table 1 shows balance for age, sex, and swimming time and distance per week, but for both sexes.
Blinding of participants and personnel (performance bias)	Low risk	See above.
Blinding of outcome assessment (detection bias)	Low risk	See above.
Incomplete outcome data (attrition bias)	Low risk	"Of the 42 participants initially recruited to the trial, three dropped out, all from the placebo group. One ... immediately after the study began, and two withdrew from competitive swimming ..." (across both males and females).
Selective reporting (reporting bias)	Low risk	Incidence of colds, duration of colds and severity of colds reported.
Vitamin C and placebo indistinguishable?	Low risk	"identical in appearance" (p 60).
Contamination	Unclear risk	There was insufficient reporting to enable assessment.

Constantini 2011M Males

**Methods**	Double-blind RCT. Duration 3 winter months.<br>This comparison is **male** swimmers.<br>See Constantini 2011F.
**Participants**	Israel. **Male** competitive adolescent swimmers with a training volume of at least 15 h/week.<br>Mean age over both sexes 14 y (12 to 17 y).<br>"Exclusion criteria were the presence of chronic health conditions and the use of prescription medications or dietary supplementations of any kind" (p 60).<br>Mean age over both sexes 14 y (12 to 17 y).<br>Vitamin C 12; placebo 10.
**Interventions**	See Constantini 2011F
**Outcomes**	See Constantini 2011F
**Notes**	See Constantini 2011F

Risk of bias table

Bias	Authors' judgement	Support for judgement
Random sequence generation (selection bias)	Low risk	See Constantini 2011F
Allocation concealment (selection bias)	Low risk	See Constantini 2011F
Baseline balance	Low risk	See Constantini 2011F
Blinding of participants and personnel (performance bias)	Low risk	See Constantini 2011F
Blinding of outcome assessment (detection bias)	Low risk	See Constantini 2011F
Incomplete outcome data (attrition bias)	Low risk	See Constantini 2011F
Selective reporting (reporting bias)	Low risk	See Constantini 2011F
Vitamin C and placebo indistinguishable?	Low risk	See Constantini 2011F
Contamination	Unclear risk	See Constantini 2011F

Himmelstein 1998

**Methods**	Double-blind RCT. Duration 3 months (Jul-Oct 1994).<br>https://www.asep.org/asep/asep/jan9.htm (1998 trial report)<br>https://www.mv.helsinki.fi/home/hemila/CC/Himmelstein_1998.htm (a copy)<br>https://www.proquest.com/docview/304251290/abstract (Thesis 1996 describing the trial in more detail).<br>The parallel trial with runners was excluded, see Notes.
**Participants**	US sedentary people; friends and coworkers of Duke City Marathon runners in Albuquerque.<br>"Subjects were restricted to no more that 200 mg/day vitamin C self-supplementation during the study period" (Report 1998: Methods).<br>Age mean 44 y (22 to 65 y) (Thesis 1996: Table 6).<br>Males 65%.<br>Vitamin C 23; placebo 25.
**Interventions**	1 g/d vitamin C for 3 months.<br>"vitamin C or placebo tablets were distributed to the study subjects at baseline which was 2 months prior to the marathon and prior to any study intervention. Subjects were restricted to a maximum of 200 mg per day of self-supplementation with vitamin C" (Thesis 1996: p 60)<br>"two tablets of either vitamin C (500 mg/tablet) or placebo (similar looking and tasting tablets containing lactose) (Hoffman-La Roche, Nutley, NJ) each morning with breakfast" (Report 1998: Methods).
**Outcomes**	**Total duration of cold symptoms:**<br>Common cold duration (Thesis 1996: Table 8; Report 1998: Table 3).<br>**Common cold severity outcome:**<br>Common cold severity (Thesis 1996: Table 8; Report 1998: Table 3).
**Notes**	A parallel trial with marathon runners was carried out, but it was excluded from our analysis, because the drop-out rate was very high and divergent in the trial arms. 52 runners started in 2 groups, but 42% (22/52) of the vitamin C group, and 75% (38/52) of the placebo group dropped out during the trial (P = 0.003).<br>Funding: "Vitamin C (500 mg) and placebo supplements were obtained at no charge from Hoffman-La Roche" (Thesis 1996: p 60).<br>"The authors gratefully acknowledge Hoffman-La Roche for providing the study supplements ...<br>This study was funded in part by a grant from the University of New Mexico Clinical Research Center" (Report 1998: Acknowledgments).

Risk of bias table

Bias	Authors' judgement	Support for judgement
Random sequence generation (selection bias)	Low risk	"Each subject was assigned a number upon entry into the study and was randomly assigned into treatment or placebo group by a computer generated randomization of the assigned numbers. Separate<br>randomization lists were maintained for runners and for sedentary subjects." (Thesis 1996: p 60),
Allocation concealment (selection bias)	Low risk	See above and "The study was conducted as a double-blind ... " (Thesis 1996: p 60).
Baseline balance	Unclear risk	Baseline balance not demonstrated.
Blinding of participants and personnel (performance bias)	Low risk	"The study was conducted as a double-blind ... " (Thesis 1996: p 60).
Blinding of outcome assessment (detection bias)	Low risk	Blinded "subjects were also instructed to complete a respiratory symptom report sheet on each day that they had a runny nose, cough, or sore throat" (Thesis 1996: p 60).
Incomplete outcome data (attrition bias)	Unclear risk	The dropout rates were 45% (19/42) in the vitamin C group and 44% (20/45) in the placebo group. (Thesis 1996: p 59; Report 1998: Methods). This level is not low, but it is equal in both groups. "two subjects, both VS [vitamin C sedentary/non-runners], had taken less than 60% of the study supplements. This finding was vastly different from the remainder of the study subjects and a post-hoc decision was made to drop them from the analysis" (Report 1998: Results.
Selective reporting (reporting bias)	Low risk	Incidence of colds, duration of colds and severity of colds reported.
Vitamin C and placebo indistinguishable?	Low risk	"Placebo (similar looking and tasting tablets containing lactose)" (Report 1998: Methods).<br>"Subjects were queried regarding which treatment they believed they were taking ... In the sedentary group no significant differences were found between actual and believed treatment ..."

		(Report 1998: p Results).
Contamination	High risk	Estimated pre-supplementation vitamin C intake in the placebo group was 227 mg/d, and in the vitamin C group 312 mg/d (Report 1998: Table 2; Thesis 1996: Table 15).

Ludvigsson 1977L Large

**Methods**	Double-blind RCT. Duration 3 months (Sep-Dec 1973).<br>This is the **Large** trial, which was carried out after the Pilot trial (Ludvigsson 1977P).<br>https://doi.org/10.3109/inf.1977.9.issue-2.07<br>https://www.researchgate.net/publication/22256528<br>https://www.academia.edu/69567236
**Participants**	Swedish school children.<br>Age mean 9.3 y.<br>Males 316, females 299.<br>Vitamin C 304; placebo 311.
**Interventions**	1 g/d vitamin C for 3 months.<br>Placebo contained 10 mg/d vitamin C.<br>"fizzy tablet which contained 1000 mg vitamin C; in the other group the fizzy tablet looked and tasted the same" (p 91).
**Outcomes**	**Total duration of cold symptoms:**<br>Duration of the common cold (Table V, p 95): "Cold symptoms from the nose (runny nose and/or sneezing and/or stuffed nose), sore throat and cough: The number of days with a cold were counted as a continuous period if they were noted down as symptom-free for up to a maximum period of 2 days at a time" (p 92).<br>**Common cold severity outcome:**<br>Duration of days off school (Table V, p 95).<br>Ludvigsson also reported outcome duration for "upper respiratory tract infection" (URTI). The incidence of the "common cold" was substantially greater than the incidence of URTI and, given the goal of our study, we used the duration of the common cold as the duration of total common cold symptoms.
**Notes**	Funding: not reported

Risk of bias table

Bias	Authors' judgement	Support for judgement
Random sequence generation (selection bias)	Low risk	"Every class was divided at random into two groups" (p 91).
Allocation concealment (selection bias)	Low risk	"Both studies were carried out totally double blind" (p 92).<br>At the end of the study "the code used [was] decoded" (p 92).
Baseline balance	Low risk	Table I shows balance for age and sex.<br>"Background variables were divided at random so that the groups became nearly identical (Table II)" (p 91).
Blinding of participants and personnel (performance bias)	Low risk	See above
Blinding of outcome assessment (detection bias)	Low risk	See above
Incomplete outcome data (attrition bias)	Low risk	Of the 642 children who started in the main study 27 dropped out (p 93). The dropout rate due to suspected side effects was 0.3% (1/304) in the vitamin C group compared with 0.3% (1/311) in the placebo group.
Selective reporting (reporting bias)	Low risk	Extensive reporting in Table V: Incidence of colds, duration of colds and severity of colds.
Vitamin C and placebo indistinguishable?	Low risk	"fizzy tablet which contained 1000 mg vitamin C; in the other group the fizzy tablet looked and tasted the same".
Contamination	High risk	Placebo contained 10 mg/d vitamin C, which is low in our view.<br>However, the dietary vitamin C intake in the placebo group seems to have been high. There was only a small difference in serum vitamin C levels between those who received 10 mg/d and those who received 1 000 mg/d vitamin C (86.4 and 107.0). Furthermore, there was no difference in the leucocyte vitamin C levels (17.0 and 17.4, resp.) (p 94).

Ludvigsson 1977P Pilot

**Methods**	Double-blind RCT. Duration 7 weeks (Mar-Apr 1973).<br>**Pilot** study to Ludvigsson 1977L Large, see Ludvigsson 1977L.
**Participants**	Swedish school children.<br>Males 83, females 75.<br>Mean age 10 y.<br>Vitamin C 80; placebo 78.
**Interventions**	1 g/d vitamin C for 3 months.<br>Placebo contained 30 mg/d vitamin C.<br>"fizzy tablet which contained 1000 mg vitamin C; in the other group the fizzy tablet looked and tasted the same".
**Outcomes**	See Ludvigsson 1977L.
**Notes**	Pilot study to Ludvigsson 1977L.<br>Funding: not reported.

Risk of bias table

Bias	Authors' judgement	Support for judgement
Random sequence generation (selection bias)	Low risk	See Ludvigsson 1977L.
Allocation concealment (selection bias)	Low risk	See Ludvigsson 1977L.
Baseline balance	Low risk	See Ludvigsson 1977L.
Blinding of participants and personnel (performance bias)	Low risk	See Ludvigsson 1977L.
Blinding of outcome assessment (detection bias)	Low risk	See Ludvigsson 1977L.
Incomplete outcome data (attrition bias)	Low risk	Of the 172 children who started in the pilot study 14 dropped out (p 93). The dropout rate due to suspected side effects was 1% (1/80) in the vitamin C group compared with 3% (2/78) in the placebo group.
Selective reporting (reporting bias)	Low risk	See Ludvigsson 1977L.
Vitamin C and placebo indistinguishable?	Low risk	See Ludvigsson 1977L.
Contamination	High risk	Placebo contained 30 mg/d vitamin C. See also Ludvigsson 1977L about comparison of serum and leucocyte vitamin C levels.

Miller 1977

**Methods**	Double-blind RCT. Duration 5 months beginning in Nov 1974.<br>Identical twins.<br>The twin pairs were separated by body weight into three dosage groups receiving 0.5 g, 0.75 g or 1 g ascorbic acid daily (p 248).<br>We included "high body weight" twins administered 1 g/d vitamin C.<br>https://doi.org/10.1001/jama.1977.03270300052006<br>https://doi.org/10.1001/jama.1977.03280100021008 (Discussion 1)<br>https://doi.org/10.1001/jama.1977.03280100021009 (Discussion 2)<br>https://pubmed.ncbi.nlm.nih.gov/569316 (1978 report)<br>https://www.mv.helsinki.fi/home/hemila/CC/Miller_1978_ch.pdf
**Participants**	US school children, ranging in age from 6 to 15 years. 12 twin pairs (5 boy pairs, 7 girl pairs) "high body weight".
**Interventions**	1 g/d vitamin C for 3 months.<br>The placebo capsules contained starch (p 248).<br>Placebo contained 50 mg/d vitamin C.
**Outcomes**	**Total duration of cold symptoms:**<br>Duration of the common cold (p 250; Table 3).<br>**Common cold severity outcome:**<br>Severity of the common cold (p 250; Table 3).
**Notes**	SD for duration was not published and it was calculated from the SE for the paired difference, see Additional file.<br>Funding: The tablets were supplied by Eli Lilly and Co., Indianapolis.

Risk of bias table

Bias	Authors' judgement	Support for judgement
Random sequence generation (selection bias)	Low risk	"Within a twin pair, the assignment to the treatment group was random" (p 248).
Allocation concealment (selection bias)	Low risk	"double-blind"; "The code was not broken until after the analysis of symptom data had been completed" (p 248).
Baseline balance	Low risk	Twins
Blinding of participants and personnel (performance bias)	Low risk	See above; and<br>"...four mothers acknowledged tasting the contents of the capsules. We cannot exclude the possibility that ... recognized the vitamin C by taste and ...may have influenced their subjective symptom ratings" (p 251).
Blinding of outcome assessment (detection bias)	Low risk	See above, and<br>"...four mothers acknowledged tasting the contents of the capsules. We cannot exclude the possibility that ... recognized the vitamin C by taste and ...may have influenced their subjective symptom ratings" (p 251).
Incomplete outcome data (attrition bias)	Low risk	1 pair of twins was omitted from the analysis because of incomplete data (p 249).<br>Because of the pairing, the dropout of a pair cannot generate a systematic bias between the groups.
Selective reporting (reporting bias)	Low risk	Incidence of colds, duration and severity of colds, and days in bed were reported.
Vitamin C and placebo indistinguishable?	Unclear risk	"The capsules contained 250 mg vitamin C or starch" (p 248).<br>"Four mothers acknowledged tasting the contents of the capsules ... cannot exclude the possibility ... that they recognized the vitamin C by taste" (p 251).
Contamination	High risk	Before the trial the placebo group excreted on average 314 mg/d vitamin C in urine and the daily intake must have been much higher (p 249; Table 2).<br>In addition, urinary vitamin C level of placebo group boys increased from baseline level of 319 mg/d to the trial level of 430 mg/d suggesting that some twins may have swapped their tablets. (p 249, Table 2)

Pitt 1979

**Methods**	Double-blind RCT. Duration 8 weeks (Oct-Dec 1974).<br>https://doi.org/10.1001/jama.1979.03290350028016<br>https://www.researchgate.net/publication/22773196<br>https://www.academia.edu/78271637
**Participants**	US male marine recruits.<br>Age mean 18.5 y.<br>Vitamin C 331; placebo 343.
**Interventions**	2 g/d vitamin C for 2 months.<br>"the placebo tablets were formulated from citric acid and were indistinguishable in appearance and taste from the vitamin C tablets" (p 908).<br>"Each recruit was instructed to take two tablets each morning and two each evening, and pill taking was supervised and observed by the drill instructors in each platoon" (p 908)
**Outcomes**	**Total duration of cold symptoms:**<br>Common cold duration (p 910; Table 2).<br>**Common cold severity outcome:**<br>Common cold severity (p 910; Table 2).
**Notes**	SD for duration and severity were not published.<br>The SD for duration was imputed as SD = mean.<br>The SD for severity was calculated from the reported P-value, see Additional file.<br>The severity of colds was classified on a numerical rating from 1 to 4. Since the minimum of their scale was 1, for our analysis we rescaled the severity levels to the range 0 to 3.<br>Funding: study was supported in part by US Navy and Hoffmann-LaRoche Inc supplied the tablets.

Risk of bias table

Bias	Authors' judgement	Support for judgement
Random sequence generation (selection bias)	Low risk	"assigned randomly ... from a list of consecutive numbers randomized in pairs. Randomization was carried out by individual recruits within each platoon" (p 908).
Allocation concealment (selection bias)	Low risk	"Neither the recruits or drill instructors nor the physicians and corpsmen who treated the recruits were aware of which pill any individual recruit was taking" (p 908).
Baseline balance	Low risk	Table 1 shows balance for age, race, previous medical history, previous cold history, work days lost per year (p 909).
Blinding of participants and personnel (performance bias)	Low risk	See above
Blinding of outcome assessment (detection bias)	Low risk	See above
Incomplete outcome data (attrition bias)	Low risk	Of the 862 recruits who began taking the pills, 64 (34 vitamin C, 30 placebo) were removed from their platoons. An additional 123 recruits (64 vitamin C, 59 placebo) were excluded from the final analysis because they did not continue to take their pills for the full study period (p 909).<br>The dropout rates were 22.8% (98/429) in the vitamin C group, compared with 20.6% (89/432) in the placebo group.
Selective reporting (reporting bias)	Low risk	Incidence of colds, duration of colds and severity of colds reported.
Vitamin C and placebo indistinguishable?	Low risk	"the placebo tablets were formulated from citric acid and were indistinguishable in appearance and taste from the vitamin C tablets" (p 908).
Contamination	Unclear risk	After 6 wk of vitamin C taking, the difference between placebo and vitamin C groups was not substantial in whole-blood vitamin C level (52 vs 77 μM [0.91 versus 1.36 mg/dL]) (p 909).<br>The then current recommended vitamin C intake (60 mg/d) is predicted to lead to plasma level about 20 μM (Levine et al. PNAS 1996;93:3704-3709), which indicates rather high intake of vitamin C from foods during the trial.

Ritzel 1961

**Methods**	Double-blind RCT. Duration about 1 week.<br>https://pubmed.ncbi.nlm.nih.gov/13741912<br>https://doi.org/10.5281/zenodo.6546378 (English translation from German, and the German original) https://doi.org/10.1001/jama.1976.03260370018017<br>(1976 Letter in JAMA about the methods).<br>Biography of Ritzel:<br>https://doi.org/10.5281/zenodo.7965598<br>Pauling's summaries of the RCT:<br>https://scarc.library.oregonstate.edu/coll/pauling/rnb/33/33-030.html<br>https://scarc.library.oregonstate.edu/coll/pauling/rnb/33/33-033.html
**Participants**	Children attending two 5 to 7 day long ski camps in Swiss Alps.<br>Vitamin C 139; placebo 140.
**Interventions**	1 g/d vitamin C for 1 week.<br>Placebo not described.<br>"Each morning such a tablet was distributed to each of the subjects and taken under supervision. There was no opportunity for the subjects to exchange tablets" (Translation).
**Outcomes**	**Total duration of cold symptoms:**<br>Duration of all colds (p 65 and p 66, Tabelle 1).<br>**Common cold severity outcome:**<br>Duration of constitutional symptoms (p 65 and p 66, Tabelle 1).
**Notes**	The SD for duration was not published and the SD was calculated from the reported P value, setting the ratio of SD in the vitamin C and placebo groups equal with the ratio of mean outcomes in the vitamin C and placebo groups, see Additional file.<br>The SD for the duration of constitutional symptoms was estimated, see Additional file.<br>Funding: tablets were supplied by Hoffmann-LaRoche, Basel.

Risk of bias table

Bias	Authors' judgement	Support for judgement
Random sequence generation (selection bias)	Low risk	"In one original package for each participant, to each of which was assigned a serial number, either 1.0 g of vitamin C or of a placebo was placed" (Translation).<br>"The children were randomly separated into two groups" (1976: p 1108).
Allocation concealment (selection bias)	Low risk	"self-controlling double-blind design" and "The study was double-blinded, neither the study participants nor the camp doctors were aware of the set up of the study" (Translation).<br>"Neither test subjects nor investigators knew whether the children got placebo or vitamin C" (1976: p 1108).
Baseline balance	Unclear risk	Baseline balance not demonstrated.
Blinding of participants and personnel (performance bias)	Low risk	See above.
Blinding of outcome assessment (detection bias)	Low risk	See above and "Professionals who had absolutely no connection with personnel involved in the study decoded and statistically evaluated the study results" (Translation).
Incomplete outcome data (attrition bias)	Low risk	Short study in a camp.
Selective reporting (reporting bias)	Low risk	Incidence of colds and duration of all colds and severe symptoms were reported.
Vitamin C and placebo indistinguishable?	Low risk	"The placebo was indistinguishable from the 1-gm ascorbic acid tablet" (1976: p 1108).
Contamination	Low risk	In our view, it seems highly unlikely that the dietary vitamin C intake was high in early 1960 in a skiing camp in Swiss Alps.

Sabiston 1974

**Methods**	Double-blind RCT. Duration 2 to 3 weeks.<br>https://doi.org/10.5281/zenodo.7303680
**Participants**	Canadian male military recruits during subarctic winter exercises.<br>Age mean 25 y (17 to 47 y).<br>Vitamin C 56; placebo 56.
**Interventions**	1 g/d vitamin C for 2 to 3 weeks.<br>Placebo not described in the report.<br>"tent-group commanders who carried with them the supply of pills for their own tent. Two pill vials were provided for each tent, one containing Vitamin C and one containing placebo. Each vial contained the names of the men who were to receive the respective pills. Pills were dispensed twice a day, once with the morning meal and once with the evening meal" (p 4).<br>Personal communication from Manny Radomski (email 12 September 2009):<br>"The Vit C and Placebo were in identical capsules, so taste did not enter into the equation We obtained the empty Placebo capsules from the Vit C supplier and then we filled the Placebo capsules in the lab. Pretty sure we used Sucrose".<br>"In our pre-briefing to the troops, we believe that we told the troops that they would all be getting Vitamin C but at different doses".<br>We (HH and EC) do not consider that sucrose is an ideal placebo, but we do not consider that it is a severe flaw.
**Outcomes**	**Total duration of cold symptoms:**<br>Duration of nasal and throat/chest symptoms during colds (p 6: Table 6).<br>**Common cold severity outcome:**<br>Duration of constitutional symptoms during colds (p 6: Table 7).
**Notes**	We received personal communication from Manny Radomski (email 12 September 2009), see extracts in this summary.<br>Funding: Canadian Army.

Risk of bias table

Bias	Authors' judgement	Support for judgement
Random sequence generation (selection bias)	Low risk	"Men in each tent were assigned randomly" (p 4).<br>Personal communication from Manny Radomski (email 12 September 2009):<br>"we did assign people randomly. We had the names of people beforehand but we assigned them randomly and we provided their names on the pill vials. The Tent Group Commander was responsible for distributing the pills and recording the distribution. He did NOT know what was in the vials".
Allocation concealment (selection bias)	Low risk	See above and<br>Personal communication from Manny Radomski (email 12 September 2009):<br>"While we pre-assigned Vit C and Placebo randomly, we did not break the code until after the trial. Two labelled vials were provided to the Tent Group Commanders but the Tent Group Commanders did NOT know what was in the vials. They just knew who to give the pills to. We did not label the vials Vit C and Placebo but, as I recall, we assigned a Number to the Vials which was coded to Vit C or Placebo".<br>"We did not 'break the code' until after all cards had been assessed".
Baseline balance	Low risk	Table 2 shows that age and common cold history were balanced.
Blinding of participants and personnel (performance bias)	Low risk	See above.
Blinding of outcome assessment (detection bias)	Low risk	See above and<br>"We collected the data by symptoms on T-Scan cards. We then came up with a 'scoring' system to define a cold, totally independent of examining any data. Then, three of us sat down and assigned 'scores' to each card without any knowledge, at all, with respect to Vit C or Placebo" (email Radomski 12 September 2009).
Incomplete outcome data (attrition bias)	Low risk	Short study in military conditions.

Selective reporting (reporting bias)	Low risk	Incidence of colds, duration of nasal and throat/chest colds, and severity of colds were reported.
Vitamin C and placebo indistinguishable?	Low risk	"Vitamin C and placebo were in identical capsules, so taste did not enter into the equation... In our pre-briefing to the troops, we believe that we told the troops that they would all be getting vitamin C but at different doses" (email Radomski 12 September 2009).
Contamination	Low risk	"it was determined that the RP-4 rations (1970-71) on which the men were living, apparently provided a maximum of 37–41 mg Vitamin C per day in a single fruit-drink mix" (p 4).<br>"The whole-blood ascorbate levels of individuals receiving a Vitamin C supplement were increased well above normal (100–150%)" (p 8).
